# A randomised controlled trial of an intervention to increase the implementation of a healthy canteen policy in Australian primary schools: study protocol

**DOI:** 10.1186/s13012-014-0147-3

**Published:** 2014-10-11

**Authors:** Luke Wolfenden, Nicole Nathan, Christopher M Williams, Tessa Delaney, Kathryn L Reilly, Megan Freund, Karen Gillham, Rachel Sutherland, Andrew C Bell, Libby Campbell, Serene Yoong, Rebecca Wyse, Lisa M Janssen, Sarah Preece, Melanie Asmar, John Wiggers

**Affiliations:** The University of Newcastle, Faculty of Health, School of Medicine and Public Health, Newcastle, NSW Australia; Hunter New England Population Health, Newcastle, NSW Australia; Hunter Medical Research Institute, Newcastle, NSW Australia; The George Institute for Global Health, Sydney, NSW Australia

**Keywords:** Healthy eating, Canteens, Schools, Policy, Nutrition, Children, Implementation

## Abstract

**Background:**

The implementation of healthy school canteen policies has been recommended as a strategy to help prevent unhealthy eating and excessive weight gain. Internationally, research suggests that schools often fail to implement practices consistent with healthy school canteen policies. Without a population wide implementation, the potential benefits of these policies will not be realised. The aim of this trial is to assess the effectiveness of an implementation intervention in increasing school canteen practices consistent with a healthy canteen policy of the New South Wales (NSW), Australia, government known as the ‘Fresh Tastes @ School NSW Healthy School Canteen Strategy’.

**Methods/design:**

The parallel randomised trial will be conducted in 70 primary schools located in the Hunter region of New South Wales, Australia. Schools will be eligible to participate if they are not currently meeting key components of the healthy canteen policy. Schools will be randomly allocated after baseline data collection in a 1:1 ratio to either an intervention or control group using a computerised random number function in Microsoft Excel. Thirty-five schools will be selected to receive a multi-component intervention including implementation support from research staff, staff training, resources, recognition and incentives, consensus and leadership strategies, follow-up support and implementation feedback. The 35 schools allocated to the control group will not receive any intervention support as part of the research trial. The primary outcome measures will be i) the proportion of schools with a canteen menu that does not contain foods or beverages restricted from regular sale (‘red’ and ‘banned’ items) and ii) the proportion of schools where healthy canteen items (‘green’ items) represent the majority (>50%) of products listed on the menu. Outcome data will be collected via a comprehensive menu audit, conducted by dietitians blind to group allocation. Intervention effectiveness will be assessed using logistic regression models adjusting for baseline values.

**Discussion:**

The proposed trial will represent a novel contribution to the literature, being the first randomised trial internationally to examine the effectiveness of an intervention to facilitate implementation of a healthy canteen policy.

**Trial registration:**

Australian New Zealand Clinical Trials Registry ACTRN12613000311752

**Electronic supplementary material:**

The online version of this article (doi:10.1186/s13012-014-0147-3) contains supplementary material, which is available to authorized users.

## Background

Researchers in developed countries including the United Kingdom (UK), United States (US) and Australia report that children fail to consume sufficient serves of fruits and vegetables and overconsume energy-dense, nutrient-poor foods and beverages increasing their risk of excessive weight gain and future chronic diseases [1–4]. Schools represent a valuable setting to improve child diet as they provide almost universal access to children on a continuous and intensive basis during crucial phases in their development of dietary habits [5]. A recent systematic review found that school policies or guidelines that focussed on increasing the availability of healthy products on the school menu, placing restrictions on the availability of unhealthy foods sold at schools or competitively pricing healthier foods significantly increased the sales of healthy products and improved child dietary intake [6]. As such, guidelines and policies governing the availability of foods in school food services, canteens and kiosks have also been recommended by the World Health Organization (WHO) and introduced by governments, internationally, including in Australia [7–11].

Despite the benefits of implementing school nutrition policies and guidelines, international research suggests that most schools fail to implement such initiatives, even when schools are obliged to do so [9–13]. A number of barriers have been suggested to impede implementation of school nutrition policies and guidelines including a lack of skill in product classification, a lack of support from parents and the school community, concerns regarding canteen profitability, a lack of canteen manager knowledge and awareness and a lack of resources [12,14]. Unless implementation support is provided to schools to overcome these barriers, the potential benefits of healthy canteen policies on public health nutrition will not be realised [15].

The aim of this research trial is to assess the effectiveness of an implementation intervention in increasing canteen practices consistent with the healthy canteen policy of the New South Wales (NSW) Government of Australia, known as the ‘Fresh Tastes @ School NSW Healthy School Canteen Strategy’. It is hypothesised that, relative to a no intervention control group, at post-intervention,

i) there will be a 35% absolute increase in the proportion of schools with a canteen menu that does not contain foods or beverages (‘red’ and ‘banned’) restricted for sale under the healthy canteen policy.
ii) there will be a 35% absolute increase in the proportion of schools where healthy canteen items represent >50% of products listed on the canteen menu—as encouraged by the healthy canteen policy.

## Methods

This study has received Australian Nationally Competitive Research Grant funding (Additional file 1) and was approved by the Hunter New England Human Research Ethics Committee (Additional file 2). The research is a collaboration between The University of Newcastle, Hunter New England Local Health District and the Australian Research Council (Additional file 3). The study methods have been reported in accordance with the CONSORT statement (Additional file 4).

### Context

In 2005, the NSW government launched the Fresh Tastes @ School NSW Healthy School Canteen Strategy as a key component of its action plan to prevent childhood obesity [16]. The strategy used a traffic light system to classify foods and beverages sold by schools as ‘red’, ‘amber’ or ‘green’ based on their nutritional properties [8]. The strategy was adopted as part of the Department of Education and Communities policy in NSW requiring all government schools to remove unhealthy foods and beverages—those classified as ‘red’—from regular sale at school canteens. Furthermore, schools were encouraged to ‘fill the menu’ with items classified as ‘green’ and not let items classified as ‘amber’ dominate the menu.

Items classified as ‘red’ based on the healthy canteen policy are those that lack adequate nutritional value and can contribute to excessive energy intake [8]. They are high in saturated fat and/or added sugar and/or salt and include sugar-sweetened drinks (banned), chocolate-coated premium ice creams, confectionary and deep-fried foods. Commercial hot foods and snacks, pastries, ice creams, savoury snack foods, sweet biscuits and cakes can also be classified as ‘red’ items if they do not meet the nutrition criteria described in Table 1. ‘Amber’ items are considered to have some nutritional value and moderate levels of saturated fat and/or added sugar and/or salt [8]. If consumed in large amounts, however, these foods can contribute to excess energy intake. ‘Amber’ items include full fat dairy products, processed meats, sauces, spreads, refined breakfast cereals, most flavoured waters, diet soft drink and ≥99% fruit juice over 200 ml. Amber items are also commercial hot foods and snacks, pastries, ice creams, savoury snack foods, sweet biscuits and cakes that do meet the criteria described in Table 1. ‘Green’ items are foods that are considered to be good sources of nutrients and contain low amounts of saturated fat and/or added sugar and/or salt [8]. They include fruits, vegetables and legumes, reduced fat dairy products, wholegrain cereals, grains such as pasta, rice and bread, lean red meat, fish, skinless poultry and alternatives, as well as small serves of ≥99% fruit juice.

**Table 1:** **Fresh Tastes @ School classification criteria for ‘red’ items [** 8 **]**

	**Food content**			
	**Energy (kj)**	**Saturated fat (g)**	**Sodium (mg)**	**Fibre (g)**
**Hot food assessed per 100 g**				
Savoury pastries, pasta, pizzas, over-baked potato products, spring rolls, fried rice and noodles	>1,000	>5	>400	
Crumbed and coated foods, e.g. patties, ribs, chicken products and sausages/frankfurts	>1,000	>5	>700	
**Snack food/drink assessed per serve**				
Sugar-sweetened drinks and ices	>300		>100	
Snack food bars and sweet biscuits	>600	>3		<1
Savoury snack foods and biscuits	>600	>3	>200	
Ice creams, milk based ice confections and Dairy desserts	>600	>3		
Cakes, muffins and sweet pastries, etc.	>900	>3		<1.5

### Design and setting

The study will employ a randomised controlled trial (RCT) design. Primary schools with a canteen will be randomised to either an intervention group or a no intervention comparison group. The effectiveness of the intervention will be determined by comparing post-intervention differences between groups in i) the proportion of schools with a canteen menu that does not sell foods or beverages (‘red’ and ‘banned’) restricted for sale according to the healthy canteen policy and ii) the proportion of schools where healthy canteen items (‘green’) comprise more than 50% of products listed on the menu as recommended by the healthy canteen policy. Data will be collected at baseline and immediately following the implementation intervention. The trial will be conducted in the Hunter region, a geographically and socioeconomically diverse region in NSW, Australia. Children in the region frequently purchase foods high in fat, salt and sugar from their school canteen [17].

### Participants and recruitment

Publically available Department of Education and Communities’ lists of primary schools in the Hunter region will serve as the sampling frame. There are over 300 schools in the study region. Schools will be randomly selected from the list and approached to participate. Schools with a canteen will be eligible if they have *either* at least one food or beverage menu item which is restricted (‘red’ or ‘banned’) for sale; or have less than 50% menu items classified as healthy foods or beverages (‘green’ items). Schools from the Catholic Schools Office and Association of Independent Schools will be ineligible, as the healthy canteen policy is not mandated in these schools. Schools with both primary and secondary students (i.e. central schools) and schools catering exclusively for children requiring specialist care will also be ineligible. Recruitment procedures will be adapted from a review of recommendations for engaging schools in research trials [18] which has been used previously in the setting to achieve participation rates of schools of between 80–95% [19,20].

### Random allocation

**Figure 1:**
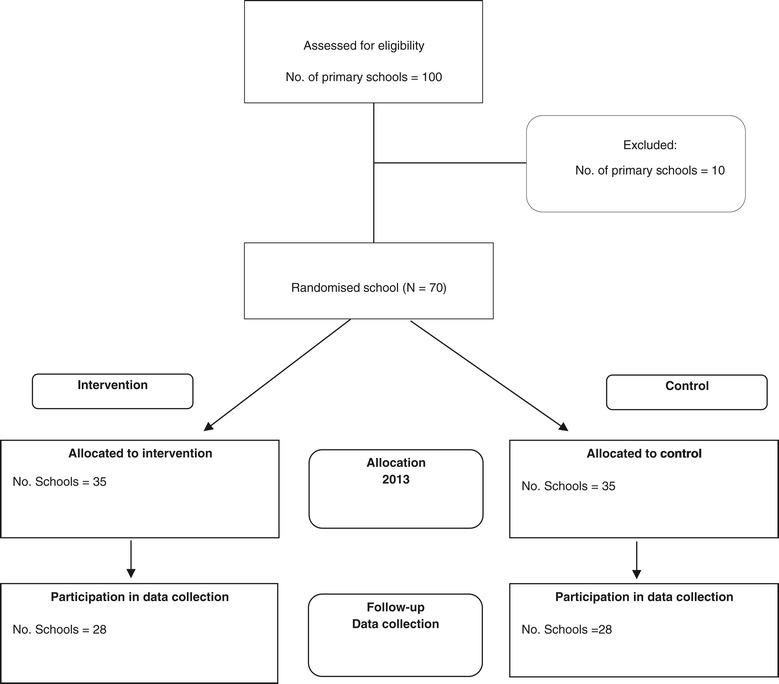
**CONSORT flowchart describing anticipated progress of participants through the trial.**

Schools will be randomly allocated after baseline data collection in a 1:1 ratio to either an intervention or control group using a computerised random number function in Microsoft Excel (see Figure 1). A block randomisation procedure will be employed to ensure group allocation is equal. The procedure will be stratified based on the socioeconomic status of a school locality given evidence that the locality may have a potential impact on the trial outcome [17].

### Intervention group

The intervention will seek to increase school implementation of the healthy canteen strategy, which is supported by a government policy.

#### Intervention components

The intervention has been designed and will be overseen by an advisory group with representation from health promotion practitioners, psychologists, dietitians, teachers, canteen managers and researchers with expertise in child obesity prevention, school-based interventions and implementation science. The multi-component implementation intervention was developed to address known barriers to the implementation of healthy canteen guidelines [12,14,21]. The selection of intervention components was guided by the theoretical domains framework [22] to address impediments to implementation. The intervention components are empirically supported by reviews of implementation and practice change interventions [23,24] and have been previously utilised to improve the health-promoting policies and practices of organisations in other community and clinical settings [25–27]. Specifically, the intervention will target the canteen manager and include the following evidence-based components delivered over a period of 12–14 months:

1. Implementation support – Each school will be allocated a support officer for the duration of the intervention to support the canteen manager with the implementation of the healthy canteen policy. Support officers will have a qualification in a health-related discipline (e.g. health promotion or dietetics). Schools will receive a bi-monthly contact from the support officer (via email, telephone or in person) throughout the intervention period. Based on principles of continuous quality improvement [28,29], each contact will aim to progress the schools’ implementation of their school canteen action plan through goal setting, action planning, self-monitoring and problem-solving.
2. Executive support [23,24] – Support officers will communicate the importance and benefits of implementing the healthy canteen policy to principals and canteen managers during school visits, support calls, training workshops and newsletters. The school principal will demonstrate executive level support for the implementation of the healthy canteen policy through the endorsement of a supportive local canteen policy and promoting this policy to teachers, parents, students and the canteen managers via staff meetings, parent meetings and newsletters. Meetings with the executive of parent representative groups within schools will be conducted to seek their input and endorsement of the process of policy implementation.
3. Consensus processes [23,24] – Consensus processes with the canteen managers and canteen staff will be conducted to reach an agreement regarding a policy implementation strategy. Support officers will assist canteen managers to develop a local canteen action plan (CAP) to co-ordinate implementation tasks.
4. Canteen manager training [23] – The canteen manager (and/ or other relevant canteen staff) will be invited to attend a 1-day (5-hr) training workshop, designed to provide education and skill development in nutrition and food classification based on the healthy canteen policy criteria, canteen stock selection, financial management, food pricing and promotion and change management. Consultations by the research staff with canteen managers indicated a high level of interest and need for such support. Training will combine didactic and interactive components including opportunities for self-assessment; practice food classification, pricing and promotion strategies; to ask questions and to receive facilitator feedback. Training incorporating both didactic and interactive elements has been found to facilitate learning [30,31]. Accredited dietitians will facilitate the training.
5. Tools and resources [23] – During the training workshop, the canteen managers will be provided with a package of resources to support them and their volunteers to implement the healthy canteen policy. Specifically, the resources will include a Fresh Tastes @ School canteen menu planner which includes a rationale and background of the healthy canteen policy and the Fresh Tastes tool kit, printed instructional materials and editable planning templates and stock management forms (on a USB), sample menu and recipe cards. In addition, schools will be able to select some basic kitchen equipment (to the value of AUD$100) to assist in the preparation of foods consistent with Fresh Tastes @ School. Written resources will be sourced from the Department of Education and Communities, Good for Kids program, the NSW government, the Healthy Kids Association and other reputable organisations. Canteen managers who do not attend the workshop will be offered a brief in-school overview of the training and provided the resource package during academic detailing visits by support officers.
6. Academic detailing [23,32] – The support officer will conduct academic detailing visits of the canteen at 1 and 3 months post canteen manager training. Consistent with the principles of academic detailing outlined by Soumerai and Avon [33], the support staff will observe the operational canteen environment, conduct a brief assessment of items for sale, provide feedback regarding canteen compliance with the healthy canteen policy, and assist with problem-solving, goal setting and action planning to improve the implementation of the policy.
7. Recognition [32] – Throughout the intervention, the schools with canteen menus assessed as compliant (>50% ‘green’ items and 0% ‘red’ or ‘banned’) with the healthy canteen policy will be sent a congratulatory letter and telephoned by the project co-ordinator. Compliant schools will also be promoted to other intervention schools using marketing strategies (outlined below).
8. Performance monitoring and feedback [23,34] – Quarterly menu reviews will be conducted, and the results will be used to compile written feedback reports to the canteen manager and school principal. The reports will include graphs displaying the progress toward implementation of the healthy canteen policy [34]. Verbal discussion of the feedback reports will occur during academic detailing visits and telephone support calls.
9. Marketing strategies [23] – Schools will receive quarterly project newsletters that will communicate the key messages of the healthy canteen policy. The newsletter will highlight case studies where schools have used innovative approaches to overcome common implementation barriers and provide information to support implementation.

#### Intervention personnel, training, supervision and monitoring

Implementation support will be provided directly to canteens by support officers with qualifications and experience in health promotion and dietetics. Support officers have experience in other similar studies and have undertaken tasks to engage opinion leaders and organisational executives, achieve research consensus, provide on-site academic detailing and ongoing support and facilitate practice adoption in schools and other community organisations [25–27]. Support officers will attend a 2-day training workshop conducted by a consultant experienced in canteen and finance management. The workshop will focus on equipping staff with sufficient knowledge and skills to deliver the intervention. Support officers will be managed by an experienced health promotion project co-ordinator and project records will be used to monitor intervention delivery according to the protocol.

### Control group and contamination

The delivery of all intervention components will be controlled by the research team and will not be provided to control group schools. During the trial period, teachers from either intervention or control group schools will be able to access NSW Government-run programs directed at supporting school promotion of healthy eating and physical activity generally [35]. Data regarding schools’ exposure to such programs and potential sources of contamination for control schools will be collected by the research team during the follow-up survey with principals and the canteen managers. Where evidence of potential bias due to contamination is apparent, sensitivity analyses will be conducted to assess the potential impact on trial outcomes.

### Data collection and measures

#### Primary trial outcomes

The primary outcomes of the trial are i) the proportion of schools with a canteen menu that does not contain foods or beverages (‘red’ and ‘banned’) restricted for sale under the healthy canteen policy and ii) the proportion of schools where healthy canteen items (‘green items’) represent >50% of products listed on the menu. At baseline and post-intervention (12–14 months following baseline), copies of canteen menus will be collected from participating schools. Each menu will be audited by two independent dietitians blind to group allocation. The menu audit will be conducted based on the procedures for menu review previously described [13]. Specifically, menu items will be classified as ‘red’, ‘amber’ or ‘green’ according to the healthy canteen policy criteria [8]. Additional information required to classify menu items which is unable to be obtained from the canteen menu will be collected by research assistants via a telephone call or canteen visit. Dietitians will use the NSW Ministry of Health guidelines to assist with item classification [8]. Discrepancies between dietitians in product classification will be resolved through discussion and consensus. Menu review and observation is considered the gold standard when measuring the school food environment [36,37].

### School characteristics and process data

#### Principal computer-assisted telephone interview (CATI)

Data regarding the operational characteristics of schools such as: the number of students and staff, existence of school nutrition policies and school participation in other school nutrition programs will be collected from the principals during a telephone survey. At follow-up, the principals from intervention schools will be asked to respond to the items assessing their involvement in supporting the implementation intervention and their perceived acceptability of the intervention.

#### Canteen manager survey

Data regarding the school canteen operational hours, number of canteen staff, canteen staff training and prior exposure to any intervention materials or resources will be collected from the canteen managers during a telephone survey. At follow-up, the canteen managers from intervention schools will complete items assessing their involvement in supporting the intervention and their perceived acceptability of the intervention via a pen and paper survey.

#### Canteen profit and losses

Consideration of both the benefits and potential unintended adverse consequences of intervention are important to assess the benefit of policy implementation [38]. Given the concerns of canteen managers regarding profitability of canteens [12,14], copies of the canteen manager’s and/or P and C treasurer’s report prepared for the school annual general meeting will be collected and compared at both intervention and control schools to assess canteen profitability. Such reports for the year prior to the intervention starting and final year of the study will be collected. Where such information is unavailable, items in the canteen manager surveys will ask managers to report the approximate canteen revenue and profit (or loss) for the year preceding baseline data collection and year during the intervention implementation.

#### Fidelity of the implementation process

Project records will be used to assess the degree to which the intervention provided by the support officers adhered to the protocol.

### Analysis and sample size

A total of 35 intervention and 35 control schools will be recruited. Primary trial outcomes will be assessed by comparing between group differences at follow-up regarding i) the proportion of schools with a canteen menu that does not contain foods or beverages (‘red’ and ‘banned’) restricted for sale under the healthy canteen policy and ii) the proportion of schools where healthy canteen items (‘green items’) represent >50% of products listed on the menu. Analyses will be performed under an intention to treat framework. Intervention effectiveness will be assessed using logistic regression models adjusting for baseline values and with all available data. Multiple imputations (including baseline observation carried forward) will be performed as part of sensitivity analysis for schools not providing follow-up data [39]. Based on previous recruitment experiences of the research team in this setting, it is anticipated that 80% of participating schools will be retained at follow-up [19]. Assuming a prevalence of 15% at follow-up in the comparison group for both primary trial outcomes, the sample will be sufficient to detect an absolute difference of 34% with 80% power and an alpha of 0.05. Subgroup analyses will be performed by school size (number of students) and measure of the socioeconomic status of the school’s geographic locality.

### Trial status

The trial is currently in the implementation stage of the intervention and has not initiated follow-up data collection.

## Discussion

The protocol provides a comprehensive description of the methods to be employed to assess the effectiveness of implementation intervention in increasing school canteen practices consistent with a healthy canteen policy. The study will provide rigorous evidence on which governments and other organisations can develop strategies to improve the nutrition environments of schools.

## Additional files

Additional file 1:
**Funding agreement between the Commonwealth of Australia as represented by the Australian Research Council and The University of Newcastle.**
Additional file 2:
**Ethics approval from Hunter New England Human Research Ethics Committee.**
Additional file 3:
**Collaborative research agreement between The university of Newcastle and Hunter New England Local Health District.**
Additional file 4:
**CONSORT 2010 checklist of information to include when reporting a cluster randomised trial.**

## Abbreviations

NSW: New South Wales
LLWatS: Live Life Well @ School
CATI: Computer-assisted telephone interview
