# Supplementary material for: A randomised controlled trial of an intervention to increase the implementation of a healthy canteen policy in Australian primary schools: study protocol
Source: Implement Sci. 2014 Oct 11;9:147. doi: 10.1186/s13012-014-0147-3 (PMC4197283; doi:10.1186/s13012-014-0147-3)
Supplement: Additional file 1: — Funding agreement between the Commonwealth of Australia as represented by the Australian Research Council and The University of Newcastle. [file 13012_2014_147_MOESM1_ESM.pdf]

Australian Government
Australian Research Council

Funding Agreement

between the

Commonwealth of Australia
as represented by the
Australian Research Council

and

The University of Newcastle

regarding funding for
Linkage Projects
to commence in
2013

Table of Clauses

Parties & Recitals 4
1. Definitions 4
2. Interpretation 6
3. Entire Agreement and Variation 7
4. Term of Agreement and Funding Period 8
5. Payment of Funding 8
6. Accuracy of Information 9
7. Use of the Funding: Activities, Facilities and Types of Work 10
8. Use of the Funding: Provision of Salaries and Relief for Teaching and for Other Duties ... 12
9. Over-expenditure by the Administering Organisation 13
10. Multi-Institutional Agreements 13
11. Partner Organisation Agreements 13
12. Default of Partner Organisation 15
13. Commencement of Project: Final Date for Commencement and Partner Organisation Written Agreements 16
14. Suspension of Project 16
15. Specified Personnel 16
16. Change of Specified Personnel 17
17. Transfer of Project or Specified Personnel 17
18. Relinquishment of a Project 19
19. Negation of Employment by the Commonwealth 19
20. Conduct of Research 19
21. Material Produced Under this Agreement, Publication and Dissemination of Research Outputs 20
22. ARC Assessments 20
23. Assets 21
24. Intellectual Property 21
25. Protection of Personal Information 21
26. Confidentiality 22
27. Acknowledgments, Publications and Publicity 23
28. Administration of the Funding 23
29. Audit and Monitoring 23
30. Access to Premises and Records 24
31. Reporting Requirements 25
32. Australian Research Integrity Committee 26

33. Copyright in Proposals and Reports 26
34. Recovery of Unspent Funds or Overpayments of Funds 27
35. Indemnity 27
36. Insurance 27
37. Dispute Resolution 27
38. Termination of the Agreement 28
39. Compliance with Law 29
40. Liaison 30
41. Applicable Law 30
SCHEDULE A 31
SCHEDULE B 32
SCHEDULE C 33

Parties & Recitals

THIS AGREEMENT is made on the 29 day of July 2013

between the

COMMONWEALTH OF AUSTRALIA ('the Commonwealth'), as represented by and acting through the Australian Research Council ('the ARC') [ABN 35 201 451 156]

and

The University of Newcastle ('the Administering Organisation').

WHEREAS:

A. The Commonwealth through the ARC operates the *Linkage Projects* scheme ('the Scheme');

B. The Commonwealth accepts that the Administering Organisation is an eligible body for the purposes of the Scheme, and the Commonwealth may provide financial assistance to support the Administering Organisation to conduct the Projects, including the approved Funding elements, being those described in Schedule A;

C. The Commonwealth is required by law to ensure the accountability of Funding and, accordingly, the Administering Organisation is required to be accountable for all Commonwealth Funding it receives under this Agreement; and

D. The Commonwealth wishes to provide Funding under the Scheme to the Administering Organisation for the purposes, and subject to the terms and conditions, set out in this Agreement.

IT IS HEREBY AGREED as follows:

1. Definitions

In this Agreement, unless the contrary intention appears:

ABN has the meaning as given in section 41 of the *A New Tax System (Australian Business Number) Act 1999.*

Administering Organisation means an Eligible Organisation which submits a Proposal for funding under the *Linkage Projects* scheme and which will be responsible for the administration of the Funding if the Project is approved for funding.

ARC means the Australian Research Council, as established under the ARC Act.

ARC Act means the *Australian Research Council Act 2001.*

ARC Fellowship means a named Fellowship position within any ARC scheme where the salary is funded wholly or partly by the ARC.

ARC website is www.arc.gov.au.

Asset includes personal, real or incorporeal property, but shall not include intellectual property.

Chief Executive Officer or CEO means the occupant of the position from time to time of the Chief Executive Officer of the ARC, or the delegate, as established under the ARC Act.

Chief Investigator or CI means a person named in the Proposal as Chief Investigator for a particular Project, or as otherwise approved by the Minister, and includes any replacement person or persons approved by the ARC in accordance with clause 16.

Commonwealth means the Commonwealth of Australia.

Confidential Information means any information which the parties agree is confidential or that is by its nature confidential.

Conflict of Interest means any conflict of interest, any risk of a conflict of interest and any apparent conflict of interest arising through a party engaging in any activity, participating in any association, holding any membership or obtaining any interest that is likely to conflict with or restrict that party participating in the Project.

Department means the Commonwealth Department of Industry, Innovation, Climate Change, Science, Research and Tertiary Education.

Eligible Organisation means an organisation listed at Appendix A of the *Linkage Projects* Funding Rules.

End of Year Report means the report described in clause 31.2.

Final Report means the report described in clause 31.4.

FTE means full-time equivalent.

Funding Agreement or the Agreement means this document.

Funding or **Funds** means the amount or amounts payable under this Agreement for each Project as specified in Schedule A.

Funding Period means the approved period set out in Schedule A for that Project, or as otherwise approved in writing by the ARC.

Funding Rules means the *Linkage Projects Funding Rules for funding commencing in 2013*.

GST has the meaning as given in section 195-1 of the *A New Tax System (Goods and Services Tax) Act 1999.*

In-kind Contribution means a contribution of goods, services, materials or time to the Project from an individual, business or organisation.

Intellectual Property includes all copyright and neighbouring rights, all rights in relation to inventions (including patent rights), plant varieties, registered and unregistered trademarks (including service marks), registered designs, Confidential Information (including trade secrets and knowhow and circuit layouts), and all other rights resulting from intellectual activity in the industrial, scientific, literary or artistic fields.

Material includes documents, equipment, software, goods, information and data stored by any means.

Minister means the Minister responsible for the administration of the ARC Act, or the Minister's delegate.

NHMRC means the National Health and Medical Research Council.

Other Eligible Organisation means an Eligible Organisation listed on a *Linkage Projects* Proposal which is not the Administering Organisation.

Other Organisation means an organisation which is listed in a *Linkage Projects* Proposal and is not an Eligible Organisation or a Partner Organisation.

Partner Investigator or PI means a person named in the Proposal as a Partner Investigator for a particular Project, or as otherwise approved by the Minister, and includes any replacement person or persons approved by the ARC in accordance with clause 16.

Partner Organisation means any company, government agency, incorporated body or other collaborating organisation, other than an Eligible Organisation or Other Organisation, named in Schedule A as a contributor to a Project, or as otherwise approved by the Minister, and includes any replacement organisation or organisations approved by

the ARC in accordance with clause 12.

Partner Organisation Cash Contribution means the cash funding for a Project provided by the Partner Organisation(s) which is transferred to and managed by the Administering Organisation in respect of a Project.

Partner Organisation In-kind Contribution means the in-kind contribution for a Project provided by the Partner Organisation(s) to the Administering Organisation in respect of a Project.

Personnel means those persons involved in the conduct of the Project.

Privacy Commissioner means the person occupying the position of Privacy Commissioner from time to time pursuant to the *Privacy Act 1988*.

Progress Report means the report described in clause 31.3.

Project means any Project as described in Schedule A or as otherwise approved by the Minister for Funding under this Agreement.

Project Leader means the first-named researcher named on a Project who is a Chief Investigator, or such other person otherwise approved by the Minister and includes any replacement person approved by the ARC in accordance with clause 16.

Proposal means a request to the ARC for the provision of funding which is submitted in accordance with the Funding Rules.

Recipient Created Tax Invoice means a tax invoice that is issued by the recipient of the goods and/or services rather than the supplier.

Research Management System (RMS) is the ARC's web-based system used to prepare and submit research proposals, assessments, rejoinders and Final Reports.

Research Office means a business unit within an Eligible Organisation that is responsible for administrative contact with the ARC regarding Proposals and Projects.

Responsible Officer means the Vice-Chancellor or other corporate head of the Administering Organisation or an officer nominated by her/him.

Scheme has the meaning given in Parties and Recitals.

Scheme Coordinator means the occupant from time to time of the position of Scheme Coordinator (*Linkage Project*s) in the ARC, or any other person to whom the administration of the *Linkage Project*s scheme may be allocated.

Special Condition means a special condition specified in the Agreement which governs the use of the Funding provided by the ARC.

Specified Personnel means the Chief Investigator(s) and Partner Investigator(s) named in Schedule A to perform the Project or as otherwise approved by the Minister.

UA means Universities Australia.

2. Interpretation

2.1 In this Agreement, unless the contrary intention appears:

(a) words in the singular number include the plural and words in the plural number include the singular;

(b) words importing a gender include any other gender;

(c) words importing persons include a partnership and a body whether corporate or otherwise;

(d) clause headings, words capitalised or in bold or italic format and notes in square

brackets ('[ ]') are inserted for convenience only, and have no effect in limiting or extending the language of provisions, except for the purpose of rectifying any erroneous cross-reference;

(e) all references to clauses are to clauses in this Agreement and all references to a schedule refer to a schedule in this Agreement;

(f) all references to dollars are to Australian dollars and this Agreement uses Australian currency;

(g) reference to any statute or other legislation (whether primary or subordinate) is to a statute or other legislation of the Commonwealth and, if it has been or is amended, replaced or supplemented, is a reference to that statute or other legislation as amended, replaced or supplemented; and

(h) where any word or phrase is given a defined meaning, any other part of speech or other grammatical form in respect of that word or phrase has a corresponding meaning.

2.2 This Agreement is subject to the ARC Act. If there is any conflict between this Agreement and the ARC Act, then the ARC Act prevails to the extent of any inconsistency.

3. Entire Agreement and Variation

3.1 This Agreement, including Schedules, the Proposal for each Project and the Funding Rules, constitutes the entire agreement between the parties and supersedes all communications, negotiations, arrangements and agreements, whether oral or written, between the parties with respect to the subject matter of this Agreement.

3.2 Notwithstanding clause 3.1, the Minister may at any time impose other requirements or conditions in connection with any Funding covered by this Agreement as provided for under the ARC Act. The Administering Organisation must as soon as possible, or as otherwise agreed in writing with the ARC, comply (or procure compliance) with any other Ministerial conditions or requirements notified by the ARC from time to time. In the event of any inconsistency between this Agreement and any such further requirements or conditions, the Administering Organisation will not be taken to have breached this Agreement where it has acted consistently with any further requirements or conditions notified under this clause.

3.3 If any part of this Agreement conflicts with any other part, that part higher in the following list shall take precedence:

(a) the terms and conditions contained in the clauses of the Agreement;

(b) the Schedules;

(c) the Funding Rules; and

(d) the Proposal.

3.4 The Administering Organisation and the Commonwealth may agree to vary this Agreement. Other than as expressly provided for in this Agreement, any variation to this Agreement must be in writing and signed by both parties.

3.5 The Administering Organisation is required to do all things incidental or reasonably necessary to give effect to this Agreement, including procuring any third parties to do such incidental or reasonably necessary things. This includes, but is not limited to the Administering Organisation's securing the agreement of all parties involved in Projects to abide by the terms and conditions of this Agreement.

4. Term of Agreement and Funding Period

4.1 This Agreement takes effect on the date it has been executed by the Administering Organisation and the ARC and continues to operate until all parties have fulfilled their obligations under this Agreement.

4.2 Subject to clauses 4.3 and 5 of this Agreement, the period of Funding is the Funding Period unless the Funding is terminated earlier in accordance with this Agreement.

4.3 The Funding Period for any Project including any element is indicative only of the intent of the Minister at the time of making the offer of Funding and is not binding on the Commonwealth. In the event that the Minister subsequently makes a determination under section 54 of the ARC Act to vary the Funding, this Agreement will continue to apply to any Project, including Awards or granted financial assistance under such a determination.

5. Payment of Funding

5.1 Subject to the provisions of the ARC Act, the terms of this Agreement and sufficient program funding being available for the Scheme, the Commonwealth shall pay the Funds to the Administering Organisation for each Project in progressive monthly instalments in accordance with Schedule A.

5.2 All Funding for a Project is subject to the following conditions:

(a) that the Project commence in accordance with clause 13;

(b) that the Project Leader listed in Schedule A lead and coordinate the Project during the Funding Period, including by having direct responsibility for the strategic decisions and the communication of results for the Project;

(c) that the Administering Organisation conduct the Project substantially in accordance with the 'Project Description' contained in the Proposal, or in the event of any variation to the Project, in accordance with the description, aims and research plan as otherwise approved by the ARC;

(d) that the Administering Organisation spend all Funds paid under this Agreement for each Project substantially in accordance with the 'Project Cost' detailed in the Proposal for that Project and any Special Conditions, or the budget as otherwise approved by the ARC and any conditions otherwise imposed by the ARC in accordance with the ARC Act, and in accordance with the requirements of this Agreement and the Funding Rules;

(e) that the Administering Organisation only receive Funding under this Agreement to which it is properly entitled;

(f) that the Administering Organisation enter into (and maintain) an agreement with each Partner Organisation that meets the requirements of clause 11;

(g) that the total Partner Organisation Contribution for a Project satisfies the minimum requirements set out in this Agreement (including in Schedule C) and the Funding Rules, unless otherwise approved by the ARC;

(h) that the Administering Organisation submit on time all reports required under this Agreement, in the form and with content satisfactory to the ARC;

(i) that progress of the Project is, in the opinion of the ARC, satisfactory;

(j) that each Chief Investigator (or any replacement person or persons approved by the ARC under clause 16.1), at all times during her/his participation in a Project, meet the criteria specified in Section 7.2 and subsection 9.5.3 (unless subsection 9.5.4 applies) of the Funding Rules, including by having direct responsibility for the strategic decisions and the communication of results for the Project;

(k) that each Partner Investigator (or any replacement persons approved by the ARC under clause 16.1), at all times during her/his participation in a Project, meet the criteria specified in Section 7.3 of the Funding Rules, including by having direct responsibility for the strategic decisions and the communication of results for the Project;

(l) that the ARC is advised in a timely manner of any and all Conflicts of Interest of parties involved in the Project which have the potential to influence or appear to influence the research and/or activities related to the Project;

(m) that the ARC is advised in a timely manner of any and all other Commonwealth funding which has been, is being, or is intended to be provided for the research and/or activities funded under this Agreement;

(n) that there is no duplication of Commonwealth funding for the research and/or activities funded for the Project under this Agreement;

(o) that the ARC is notified in writing in a timely manner if any Specified Personnel is not able to undertake the Project, or to continue to undertake the Project, and that any change in Specified Personnel is approved in accordance with clause 16;

(p) that the Administering Organisation is responsible for any and all taxation implications associated with receiving the Funds; and

(q) that the Administering Organisation comply with any other requirements or conditions imposed by the ARC in connection with any Funding covered by this Agreement.

5.3 If the Administering Organisation does not meet any one or more of the conditions listed in clause 5.2 in respect of a Project, the Commonwealth may do any or all of the following:

(a) not pay the Administering Organisation any further Funds for that Project;

(b) recover all or some of the Funds paid under this Agreement for that Project, including all unspent Funds and any Funds not spent in accordance with this Agreement; and/or

(c) vary the amount of Funding approved for that Project.

5.4 The Administering Organisation must pay to the Commonwealth the amount specified in any notice received under clause 5.3(b) within 30 days of the date of that notice.

5.5 The Commonwealth will pay to the Administering Organisation, by way of financial assistance in accordance with the ARC Act, the approved amounts set out in Schedule A, which is net of any GST which may be imposed on the supply.

5.6 Where the invoice relates to a taxable supply made under this contract, the invoice must comply with the requirement for a tax invoice as defined in the GST Act.

5.7 The Commonwealth shall have the right to vary, unilaterally, the amounts for any or all Projects.

5.8 Where the Commonwealth exercises its right under clauses 5.3 or 5.7 above, it shall inform the Administering Organisation in writing of the variation within 30 days of that variation having been made.

6. Accuracy of Information

6.1 The provision of any Funding for a Project is conditional on all information contained in the Proposal for that Project and all reports required by this Agreement from the Administering Organisation being complete, accurate and not misleading. The Commonwealth regards inaccurate and misleading information as including, but not being

limited to, claiming fictitious track records, inflating funds obtained from other sources and false claims in the publication record, e.g. describing a paper as being 'in press' or accepted even if it has only been submitted.

6.2 If the Commonwealth considers that a Proposal for a Project or any report provided under this Agreement contains incomplete, inaccurate or misleading information, the Commonwealth may by notice in writing to the Administering Organisation do any or all of the following:

(a) not pay the Administering Organisation any further Funds for that Project;

(b) recover all or some of the Funds paid under this Agreement for that Project, including all unspent Funds and any Funds not spent in accordance with this Agreement; and/or

(c) vary the amount of Funding approved for that Project.

7. Use of the Funding: Activities, Facilities and Types of Work

7.1 The Administering Organisation will ensure that each Project is carried out in accordance with this Agreement in a diligent and competent manner. In addition, each Project will be conducted in accordance with the 'Proposal Description' contained in the Proposal, or any revised budget, aims and research plan which have been submitted by the Administering Organisation and approved by the ARC.

7.2 The Administering Organisation must ensure that expenditure on each Project described in Schedule A is in accordance with the 'Proposal Description' contained in the Proposal and within the broad structure of the proposed 'Project Cost' detailed in the Proposal or any revised budget, aims and research plan submitted by the Administering Organisation which has been approved by the ARC.

7.3 The Administering Organisation must not use the Funding:

(a) for purposes specifically excluded in the Funding Rules; or

(b) for purposes specifically excluded in this Agreement, for example clauses 7.6, 7.15 and 8.1.

7.4 The Administering Organisation must ensure that Specified Personnel listed in Schedule A (or any replacement person(s) approved by the ARC under clause 16.1) have adequate time to carry out each Project and must provide the basic facilities required for each Project. Project Funding cannot be used for these purposes. Basic facilities include but are not limited to:

(a) access to a basic library collection;

(b) access to film or music editing facilities;

(c) accommodation (e.g. laboratory and office space, suitably equipped and furnished);

(d) provision of basic computer facilities such as desktop computers, portable computer devices, printers, word processing and other standard software;

(e) standard reference materials or funds for abstracting services;

(f) use of photocopiers, telephones, mail, fax, email and internet services; and

(g) web hosting.

7.5 The Administering Organisation may not apply 'bench fees' or similar fixed charges to ARC funded Projects for the provision of basic facilities as outlined in clause 7.4.

7.6 As set out in the Funding Rules, Funds may not be used for:

(a) 'bench fees' or similar laboratory access fees levied by the institution;

(b) capital works and general infrastructure costs, in whole or in part;

(c) costs not directly related to research, for example, professional membership fees, fees for patent application and holding, visas, relocation costs, insurance, and mobile phones (purchase or call charges);

(d) fees for international students or the Higher Education Contribution Scheme (HECS) and Higher Education Loan Programme (HELP) liabilities for students; and

(e) salaries and/or on-costs, in part or in whole, for CIs and PIs.

7.7 Funding is permitted for domestic and international travel costs (economy) for Chief Investigators and Partner Investigators, where provision for such was included in the Proposal and is not prohibited as a Special Condition. The ARC's funding contribution is limited to a maximum total of $50,000 over the duration of the Project.

7.8 The ARC recognises that in order to complete the project it may be necessary to obtain the expertise of a third party. The ARC will only approve Funding for the expert services of a third party if the services are deemed to be directly related to and necessary for the proposed Project. Such services include, but are not limited to:

(a) Translation services, transcription services;

(b) Workshop services, data collection and analysis services; and

(c) Purchase of bibliographical or archival material (electronic or hardcopy).

7.9 Publication and dissemination of Project outputs and outreach activity costs may be supported at up to two (2) per cent of total ARC Funding awarded to the Project. This excludes fees for patent application and holding.

7.10 Maintenance and access to infrastructure and equipment required for the Project may be supported. Funding will not be provided for infrastructure or equipment that is deemed to be for broad general use.

7.11 Salary support for research associates and assistants, technicians and laboratory attendants at an appropriate salary level, including 28 per cent on-costs, for the Administering Organisation may be supported.

7.12 Stipends for postgraduate students, in whole or in part, at an appropriate level for the Administering Organisation or the relevant industry may be supported.

7.13 Specialised computer equipment and software essential to the Project may be supported.

7.14 Field or survey research expenses (including travel) for Specified Personnel and research support personnel may be supported.

7.15 Unless otherwise approved by the ARC, the Funding must not be used to fund any research and/or activities for which other financial assistance from the Commonwealth has been, is being, or is intended to be provided.

7.16 Unless otherwise approved by the Minister, the Administering Organisation must provide the resources to undertake each Project as specified in the Proposal.

7.17 The Administering Organisation must obtain the agreement of all parties necessary to allow each Project to proceed. Evidence of agreement must be obtained from all relevant persons and organisations involved in the Project and is to be retained by the Administering Organisation. This evidence must be made available if requested by the ARC.

7.18 The Administering Organisation must not allow a Project to commence, nor Funding to be expended, until it has entered into a written partner agreement with each Partner Organisation in accordance with clause 11. The Administering Organisation must reach

agreement with each Partner Organisation and enter into a written partner agreement with each such organisation before the final date for commencement of the Project as determined in accordance with clause 13.

7.19 If any other Commonwealth funding is approved for any research and/or activity which is similar to research or activities being conducted for any Project, the ARC must be notified immediately and the ARC may consider whether or not to terminate or recover funding to the extent that it is duplicated by another Commonwealth source.

7.20 Where a PhD stipend is to be paid from Project costs:

(a) the PhD stipend per annum must be at an appropriate level for the Administering Organisation or the relevant industry;

(b) the PhD stipend recipient must be enrolled in a PhD at an Eligible Organisation as listed in Appendix A of the Funding Rules;

(c) management of candidature of PhD stipend recipients will be at the discretion of the Administering Organisation or host organisation as appropriate. This includes but is not limited to selection, appointment, periods of leave and/or suspension of candidature; and

(d) other costs of candidature will be the responsibility of the Administering Organisation or host organisation as appropriate. Costs of candidature may include, but are not limited to, costs of relocation, periods of leave and thesis production.

8. Use of the Funding: Provision of Salaries and Relief for Teaching and for Other Duties

8.1 The Funding specified in Schedule A must not be used to provide salary support for Chief Investigators or Partner Investigators.

8.2 Project payments can only commence once the Funding Agreement has been executed by both parties. Retrospective salary payments will not be paid for employment prior to the date the Funding Agreement is executed.

8.3 Funding may be used for the payment of a Partner Investigator's costs incurred because of her/his involvement in the Project, where such visits and costings were outlined in the Proposal and as a Special Condition were not prohibited.

8.4 If expenditure is incurred as allowed for in clause 8.3 above, the Administering Organisation must ensure that expenditure is in accordance with the broad structure of the 'Proposal Description' and 'Project Cost' detailed in the Proposal, or any revised Project budget, aims and research plan submitted by the Administering Organisation which are approved by the ARC.

8.5 Funding for Project costs may be used by the Administering Organisation to employ Personnel, other than the Specified Personnel, where provision for such was included in the Proposal. They may be employed full-time or part-time, as required.

8.6 The Administering Organisation must ensure that a person who is studying full-time for a postgraduate degree or other postgraduate qualification shall not be employed on a Project for more than 20 hours per week.

8.7 In respect of Personnel other than Chief Investigators or Partner Investigators, unless the ARC otherwise determines:

(a) in recruiting Personnel, the Administering Organisation shall follow its normal recruitment procedures;

(b) the provision of salaries, recreation leave, sick leave and other conditions of

employment for Personnel shall be those of the Administering Organisation; and

(c) the on-costs provisions beyond the ARC contribution of 28 per cent remain the responsibility of the Administering Organisation, e.g. extended periods of leave, severance pay, etc. must not be provided from ARC Funds.

8.8 Funding may be used by the Administering Organisation to fund the relief of Chief Investigators from teaching or other duties, where provision for such was included in the Proposal and is not prohibited as a Special Condition. The ARC's funding contribution is limited to a maximum total of $40,000 per annum, for all Chief Investigators, over the duration of the Project.

8.9 The Administering Organisation must ensure that any Personnel who are employed full-time on a Project and whose salary is provided from the Funding, shall not, without the prior agreement of the ARC, accept any remuneration whatsoever from any source other than the Administering Organisation in respect of work performed on the Project.

8.10 Notwithstanding clause 8.9, a Partner Organisation's cash contribution to the Administering Organisation for the Project may be used to raise the levels of the salaries paid to Personnel, excluding the salaries of Chief Investigators and Partner Investigators.

9. Over-expenditure by the Administering Organisation

9.1 Any Project expenditure incurred by the Administering Organisation for a Project additional to the approved amount for that Project specified in Schedule A, or as otherwise varied by the ARC, is the responsibility of the Administering Organisation. The Commonwealth will not reimburse the Administering Organisation for such costs under any circumstances.

10. Multi-Institutional Agreements

10.1 The Administering Organisation must not allow a Project to commence, nor Funding to be expended, until it has entered into an agreement with each Other Eligible Organisation in accordance with this clause 10.

10.2 Evidence of agreement must be made available to the ARC if required.

10.3 A written agreement must be entered into with an Other Eligible Organisation within 30 days of commencement of the Project and must include provisions that:

(a) outline the role and contribution, if any, of the Other Eligible Organisation;

(b) outline contributions and research undertaken by any other organisations involved on the Project;

(c) describe the Intellectual Property arrangements that apply to the outcome or results generated by the Project. Such arrangements must, unless otherwise approved by the ARC, comply with the *National Principles of Intellectual Property Management for Publicly Funded Research*; and

(d) do not impede or prevent the Administering Organisation from complying with any of its obligations under this Agreement.

10.4 The Administering Organisation will retain the written agreement, and make it available to the ARC if required.

11. Partner Organisation Agreements

11.1 The Administering Organisation must not allow a Project to commence, nor Funding to be expended, until it has entered into a written partner agreement with each Partner Organisation in accordance with this clause 11. The Administering Organisation must

reach agreement with each Partner Organisation and enter into a written partner agreement with each such organisation before the final date for commencement of the Project as determined in accordance with clause 13.

11.2 An agreement entered into with a Partner Organisation must include provisions that:

(a) outline the role and contribution of the Partner Organisation;

(b) outline the contributions and research undertaken by any other organisations involved on the Project;

(c) describe the Intellectual Property arrangements that apply to the outcome or results generated by the Project. Such arrangements must, unless otherwise approved by the ARC, comply with the *National Principles of Intellectual Property Management for Publicly Funded Research*;

(d) an assurance from the Partner Organisation that the Partner Organisation's relationship with the Administering Organisation and the Specified Personnel for the Project complies with the requirements specified in the Funding Rules, including:

i. that the Partner Organisation's relationship with the Chief Investigators on the Project would not generate or represent a Conflict of Interest, except where permitted under subsection 9.5.4 of the Funding Rules;

ii. that the Partner Organisation satisfies the requirements for a Partner Organisation stipulated in Sections 6.2 and 6.3 of the Funding Rules; and

iii. if one or more of the Partner Investigators are employees of the Partner Organisation, that the Partner Organisation will make an appropriate contribution of time and operating costs towards the Project in relation to the participation of those Partner Investigators in the Project;

(e) other than where the Partner Organisation is a Commonwealth entity which contracts as part of the Commonwealth, an indemnity from the Partner Organisation in favour of the Administering Organisation that covers any loss, liability or expense incurred or suffered by the Administering Organisation as a result of any breach of this Agreement caused by the Administering Organisation's reliance on the assurance given by the Partner Organisation in accordance with paragraph (d) above;

(f) are consistent with details contained in the Proposal, except as provided for in clause 11.5; and

(g) do not impede or prevent the Administering Organisation from complying with any of its obligations under this Agreement.

11.3 A written partner agreement entered into under this clause 11 must continue to satisfy the requirements of this clause 11 at all times during the Funding Period for the Project.

11.4 The Administering Organisation must ensure that the total Partner Organisation Contribution for a Project satisfies the requirements of this Agreement (including using the criteria set out in Schedule C) and the Funding Rules.

11.5 Unless otherwise approved by the ARC, the Administering Organisation must ensure that each Partner Organisation provides contributions as set out in the Proposal. However, if the Funding for a Project is less than the amount requested in the Proposal for that Project, the Partner Organisation Contribution may be reduced by the same proportion. In all cases, the minimum requirements for the eligible Partner Organisation Contribution as set out in this Agreement and the Funding Rules continue to apply.

11.6 The Administering Organisation must ensure the Commonwealth and each Partner Organisation is provided with timely notice of progress made on the Project.

11.7 Once the written partner agreement for a Project has been entered into by all participating Partner Organisations and the Administering Organisation, the Administering Organisation must provide to the ARC the *Partner Organisation Agreed Contribution Report*, on the relevant form made available by the ARC.

11.8 If a written agreement between a Partner Organisation and the Administering Organisation is revised in accordance with this Agreement to change the level of Partner Organisation Contribution, the Administering Organisation must provide to the ARC a revised *Partner Organisation Agreed Contribution Report*, on the relevant form made available by the ARC.

11.9 The Administration Organisation will retain the written partner agreement, and make it available to the ARC if required.

12. Default of Partner Organisation

12.1 If the Administering Organisation receives notice that a Partner Organisation wishes to withdraw its support for the Project, or reasonably believes that a Partner Organisation is in default of any of its obligations under a written partner agreement entered into between that Partner Organisation and the Administering Organisation under clause 11, the Administering Organisation must immediately notify the ARC, and may attempt to find a replacement Partner Organisation for the Project or modify remaining Partner Organisation arrangements, in accordance with the procedure outlined in clause 12.2.

12.2 Should the Administering Organisation wish to proceed with a replacement Partner Organisation or modified Partner Organisation arrangements as allowed under clause 12.1, it must complete the procedure listed below within three months from the date of notification from the Partner Organisation or from the date on which the Administering Organisation became aware that the Partner Organisation is not meeting its obligations, whichever is the earlier. By the end of the three-month period referred to in this clause:

(a) the Administering Organisation must request, in writing, the ARC's approval of a replacement Partner Organisation or modified remaining Partner Organisation arrangements which comply with the requirements for Partner Organisations and Partner Organisation Contributions specified in this Agreement and the Funding Rules;

(b) the replacement or remaining Partner Organisations must provide a written undertaking to provide (in total) a replacement contribution equivalent to that which would have been provided by the Partner Organisation in default and which satisfies the requirements of this Agreement (including using the criteria set out in Schedule C) and the Funding Rules; and

(c) the Administering Organisation and the replacement Partner Organisation or remaining Partner Organisations must enter into a written partner agreement, or amend any existing agreement (as appropriate) consistent with clause 11 to reflect the revised Partner Organisation Contribution arrangements.

12.3 The ARC may approve a replacement Partner Organisation if the replacement Partner Organisation meets the eligibility criteria as specified in the Funding Rules.

12.4 If the ARC approves a replacement Partner Organisation or modified remaining Partner Organisation arrangements proposed under clause 12.2, the Administering Organisation must within three months of the date of approval by the ARC provide to the ARC a revised *Partner Organisation Agreed Contribution Report*, on the relevant form made available by the ARC, reflecting the new approved arrangements.

12.5 To avoid doubt, the funding condition set out in clause 5.2(f) will not have been satisfied if:

(a) the ARC does not approve any replacement Partner Organisation or the modified Partner Organisation arrangements proposed under clause 12.2; or

(b) the Administering Organisation does not attempt to find any replacement Partner Organisation or modify existing Partner Organisation Contribution arrangements in accordance with clause 12.2.

13. Commencement of Project: Final Date for Commencement and Partner Organisation Written Agreements

13.1 The Project must commence by no later than 30 June 2014.

13.2 Pursuant to clause 7, the Administering Organisation must not allow a Project to commence, nor Funding to be expended, until it has entered into a written partner agreement with each Partner Organisation in accordance with clause 11. The Administering Organisation must reach agreement with each Partner Organisation and enter into a written partner agreement with each such organisation before the applicable commencement date for the Project as determined in accordance with clause 13.1.

13.3 Commencement of the Project cannot be deferred beyond the commencement date stated in clause 13.1. Requests for deferred commencement dates will not be considered. The ARC may recover Funding for any Project which has not commenced by the date specified in clause 13.1.

14. Suspension of Project

14.1 If any of the Specified Personnel on a Project is not able to perform the Project for a period or periods of time the Project may be suspended for a period or periods totalling up to 12 months. The duration of a Project may be extended for a period equal to the duration of the approved suspension(s). The Funds for the Project which would otherwise have been payable during the suspension period(s) will continue to be paid to the Administering Organisation during the suspension period(s) and the ARC will not supplement the Funds to cover any additional costs incurred as a result of the suspension or delay in finalisation of the Project.

14.2 If a proposed suspension is to commence after the first 12 months of the Project and is for six months or less, the Administering Organisation may, at its discretion, approve the suspension. The Administering Organisation must notify the ARC of the suspension in its next Progress or Final Report for the Project.

14.3 If the proposed suspension is to commence within the first 12 months of the Project or is for more than six months, the Responsible Officer must submit a *Variation of Funding Agreement* request to the Scheme Coordinator to seek the ARC's approval to suspend the Project. The ARC will approve such suspensions only if detailed written justification for the request is provided. The approval of a suspension is at the ARC's absolute discretion. If approved, the Administering Organisation must detail the suspension in its next Progress or Final Report for the Project.

14.4 Subject to clause 14.2, suspensions without written approval will be a breach of this Agreement and may result in the immediate termination of the Project.

15. Specified Personnel

15.1 The Administering Organisation shall ensure that the Specified Personnel conduct the Project in a diligent and competent manner and will comply with this Agreement.

15.2 The Administering Organisation shall provide each Specified Personnel with a copy of this Agreement within a reasonable time after the commencement of the Funding.

15.3 The Administering Organisation warrants that it has made proper inquiries of the Specified Personnel in relation to their eligibility pursuant to their eligibility to perform the Project.

15.4 The Administering Organisation must ensure that, unless otherwise approved by the ARC, all Specified Personnel meet the criteria specified in the Funding Rules for the full term of their participation in the Project.

15.5 The Administering Organisation must ensure that each of the Specified Personnel who is to work on a Project has the approval of her/his employing organisation to participate in the Project. The Administering Organisation does not require the agreement of the employing organisation of an overseas-based Partner Investigator (unless they are employed by an Eligible Organisation) to participate in the Project.

16. Change of Specified Personnel

16.1 If a Chief Investigator or Partner Investigator is at any time during the term of a Project no longer able to continue working on the Project, the Project may continue provided that:

(a) there is at least one original Chief Investigator working on the Project;

(b) all replacement Chief Investigators or Partner Investigators meet the eligibility criteria, as specified in the Funding Rules for the particular role they are to perform, for the period for which they are to perform that role;

(c) all replacement Chief Investigators must have research expertise which is commensurate with the standard of the original research team which was awarded the Project;

(d) all replacement Partner Investigators must have research and/or supervision/mentoring and/or management expertise which is commensurate with the standard of the original research team which was awarded the Project;

(e) approval is sought from the ARC for the change in Specified Personnel:

i. in writing (including with the request a copy of the proposed replacement Personnel's curriculum vitae and, where appropriate for the role they are to undertake, their research opportunity and performance evidence details); and

ii. within 3 months of the date that the Specified Personnel cease working on the Project;

(f) if replacement Chief Investigators or Partner Investigators ('New Personnel') are proposed for a Project, the Administering Organisation must obtain certifications from the New Personnel and their employers which have similar effect to those required to be obtained for the other Specified Personnel on the Project; and

(g) the change in Specified Personnel is approved, in writing, by the ARC.

16.2 If a Chief Investigator is the only Chief Investigator on a Project, and her/his involvement with the Project is to cease, Funds for the Project will be terminated.

16.3 If the Project Leader's involvement with the Project is to cease, another Investigator who was an original Chief Investigator on the Proposal may become the Project Leader.

17. Transfer of Project or Specified Personnel

17.1 The Administering Organisation must promptly notify the ARC of any Specified Personnel on a Project who moves to an Eligible Organisation other than the Administering Organisation at any time during the Funding Period for that Project.

17.2 If the transferring Specified Personnel is the Project Leader, then the Administering Organisation may seek the ARC's approval for the transfer of the Project to a new Eligible Organisation by submitting a *Variation of Funding Agreement* request outlining

arrangements for the continuation of the Project and the continued administration of the funding.

17.3 If the proposed transfer of the Project is to another Eligible Organisation, the Administering Organisation may seek the ARC's approval for the transfer of the Funding from the Administering Organisation to another Eligible Organisation ('the recipient Eligible Organisation') by submitting a *Variation of Funding Agreement* request to the ARC. The ARC shall have regard to the circumstances surrounding the proposed transfer and may approve the transfer subject to such conditions as the ARC considers appropriate. The request must provide evidence that:

(a) the following parties agree to the transfer:

i. the Administering Organisation;

ii. the Partner Organisation(s); and

iii. the recipient Eligible Organisation;

(b) the recipient Eligible Organisation and the Project's Partner Organisation(s) will enter into a written partner agreement of the type set out in clause 11.

(c) the recipient Eligible Organisation agrees to provide for the Project (including costs and in-kind financial support and facilities of commensurate quality) equivalent to that which would have been provided by the Administering Organisation and which satisfies the requirements of this Agreement and the Funding Rules;

(d) the research already developed will not be put at risk as a result of the transfer; and

(e) the transfer will further enhance the research.

17.4 When a Proposal is requesting the transfer of Funding the ARC may approve the transfer of unspent Funds and indicative Funding for the Project and any Assets as outlined in clause 23 to the recipient Eligible Organisation.

17.5 If ARC approval is granted in such circumstances to transfer the Funding (and any Assets):

(a) the Administering Organisation must:

i. agree to any variation or termination (as applicable) of this Agreement proposed by the ARC to give effect to the changed Funding arrangements;

ii. provide to the ARC, in writing, the amount of all unspent Funds for the Project and pay the ARC such unspent Funds. The ARC may then provide the unspent Funds to the recipient Eligible Organisation;

iii. report expenditure of Funding for the Project prior to the transfer in its End of Year Report and identify the transfer in the relevant column; and

iv. comply with any other directions reasonably given by the ARC to give effect to the transfer;

(b) the recipient Eligible Organisation will be required to:

i. enter into a new, or vary an existing, Funding Agreement with the ARC to give effect to the changed funding arrangements;

ii. report expenditure of Funding for the Project subsequent to the transfer in its End of Year Report and identify the transfer in that Report; and

iii. enter into a written partner agreement with the Project's Partner Organisation(s) of the type set out in clause 11.

17.6 If funding for a Project which was previously administered by another Eligible Organisation is to be transferred to the Administering Organisation, the Administering Organisation must ensure that the Project which is being transferred and the Specified

Personnel performing the Project satisfy the eligibility and accountability requirements of the Funding Rules and terms and conditions of this Agreement.

17.7 Relocation expenses associated with the transfer of any Specified Personnel will not be paid by the ARC.

17.8 The Project or any equipment purchased with either the Funding or the Project's Partner Organisation Contribution (including any equipment which comprises the Partner Organisation Contribution) must not be transferred to the recipient Eligible Organisation until ARC approval for the transfer of the Funding is granted.

18. Relinquishment of a Project

18.1 If, at any time during the Funding Period, the Project is relinquished by the Administering Organisation, Funding for the Project will be terminated. In such cases, any unspent Funding for the Project will be recovered by the ARC.

18.2 The Administering Organisation shall immediately advise the Scheme Coordinator of the relinquishment of the Project by means of submitting a *Variation of Funding Agreement* request to the ARC. The End of Year Report must also contain details regarding any Projects that have been relinquished.

19. Negation of Employment by the Commonwealth

19.1 Specified Personnel and the Administering Organisation must not represent themselves as being employees, partners or agents of the Commonwealth, or as otherwise able to bind or represent the Commonwealth.

19.2 Specified Personnel and the Administering Organisation shall not by virtue of this Agreement be or be deemed to be, employees, partners, or agents of the Commonwealth, or as having any power or authority to bind or represent the Commonwealth.

20. Conduct of Research

20.1 The Project must be conducted in accordance with any Special Conditions specified in this Agreement and with any other requirements or conditions imposed by the ARC in connection with any Funding covered by this Agreement.

20.2 The Administering Organisation must ensure that a Project under this Agreement will not proceed without appropriate ethical clearances from the relevant committees and/or authorities referred to in Schedule B or prescribed by the Administering Organisation's research rules. Responsibility for ensuring such clearances have been obtained remains with the Administering Organisation.

20.3 All parties involved in or associated with a Project are required to disclose to the ARC, and the other parties involved in the Project, any actual or potential Conflict of Interest which has the potential to influence, or appear to influence, the research and activities, publications and media reports, or requests for funding related to the Project.

20.4 If the Administering Organisation or any Specified Personnel become aware of any such actual or potential Conflict of Interest relating to any party involved in a Project, the Administering Organisation must:

(a) notify the ARC immediately of the nature and details of the Conflict of Interest; and

(b) have established processes in place for managing the actual or potential Conflict of Interest for the duration of the Project. Such processes must comply with the NHMRC/ARC/UA *Australian Code for the Responsible Conduct of Research Practice* (2007).

20.5 If the Administering Organisation or any Specified Personnel has failed to disclose a Conflict of Interest, the Commonwealth may do any of the things provided for in clause

5.3.

20.6 Each Project must, unless otherwise approved by the ARC in writing, conform to the principles outlined in the following and their successor documents:

(a) NHMRC/ARC/UA *Australian Code for the Responsible Conduct of Research* (2007);

(b) as applicable, the NHMRC/ARC/UA *National Statement on Ethical Conduct in Human Research* (2007); and

(c) as applicable, codes on animal research promulgated by the NHMRC.

21. Material Produced Under this Agreement, Publication and Dissemination of Research Outputs

21.1 The Administering Organisation must establish and comply with its own procedures and arrangements for the ownership of all Material produced as a result of any Project funded under this Agreement.

21.2 For any Material produced under this Agreement, the Administering Organisation must ensure that all Specified Personnel:

(a) take reasonable care of, and safely store, any data or specimens or samples collected during, or resulting from, the conduct of their Project;

(b) make arrangements acceptable to the ARC for lodgement with an appropriate museum or archive in Australia of data or specimens or samples collected during, or resulting from, their Project; and

(c) include details of the lodgement or reasons for non-lodgement in the Progress Reports and the Final Report for the Project.

21.3 The ARC will support publication and dissemination costs as per clause 7.9 of this Agreement.

21.4 All Proposals and ARC-funded research projects must comply with the ARC Open Access policy, which is available at: www.arc.gov.au. In accordance with this policy, the ARC requires that any publications arising from a Project must be deposited into an open access institutional repository within a twelve month period from the date of publication. The ARC strongly encourages the depositing of data arising from a Project in an appropriate publicly accessible subject and/or institutional repository.

21.5 The Final Report must justify why any publications from a Project have not been deposited in an open access institutional repository within twelve months of publication. The Final Report must outline how data arising from the Project have been made publicly accessible where appropriate.

21.6 This clause survives the expiration or earlier termination of this Agreement.

22. ARC Assessments

22.1 The Administering Organisation must ensure that, for the term of this Agreement, if requested by the ARC, Chief Investigators agree to assess up to 20 new proposals for ARC funding per annum for each year of Funding.

22.2 If the ARC determines that a Chief Investigator has failed to meet the obligation to assess proposals assigned by the ARC for assessment, the ARC may notify the Administering Organisation in writing of that failure.

22.3 If a Chief Investigator does not undertake assessment of the assigned proposals within a period specified by the ARC of the notice referred to in clause 22.2, the Administering Organisation will be considered to be in breach of this Agreement and Funding for the

relevant Projects on which the Chief Investigator is listed as Specified Personnel under this Agreement may be terminated.

23. Assets

23.1 Unless otherwise approved by the ARC, Assets purchased with Funding must be purchased for the exclusive purposes of the Project for the duration of the Funding Period.

23.2 The Administering Organisation shall establish and comply with its own procedures and arrangements for purchasing, installing, recording, maintaining and insuring all items of equipment purchased with the Funds.

23.3 The Administering Organisation shall ensure that any Personnel shall have first priority in the use and operation of equipment purchased for the Project and the Administering Organisation must, so far as is practicable, permit persons authorised by the ARC to have priority access to that equipment in preference to other persons.

23.4 Unless otherwise approved by the ARC, the ownership of any Asset purchased wholly or partly with the Funding shall be vested in the Administering Organisation, located on its campus and listed in its assets register unless:

(a) otherwise specified in the Proposal;

(b) the Project is terminated, in which case the ARC may, by notice in writing, require the transfer of any such item of equipment to the Commonwealth; or

(c) the Project is transferred to another organisation in accordance with clause 17, in which case, subject to the agreement of both the Administering Organisation and the recipient Eligible Organisation under that clause, the equipment purchased with Funds provided under this Agreement for the relevant Project may be transferred in accordance with clause 17.8.

24. Intellectual Property

24.1 The Administering Organisation must adhere to an Intellectual Property policy, approved by the Administering Organisation's governing body, which has as one of its aims the maximisation of benefits arising from research. The ARC makes no claim on the ownership of Intellectual Property brought into being as a result of the Projects for which Funding is provided.

24.2 Unless otherwise approved by the ARC, the Administering Organisation's Intellectual Property policy referred to in clause 24.1 must comply with the *National Principles of Intellectual Property Management for Publicly Funded Research* as amended from time to time.

24.3 The Administering Organisation, if it is not a Commonwealth Entity which is contracting on behalf of the Commonwealth, will indemnify the Commonwealth, its officers, employees and agents against any liability, loss, damage, costs and expenses arising from any claim, suit, demand, action or proceeding by any person, in respect of any infringement (or alleged infringement) of Intellectual Property rights by the Administering Organisation, its employees, agents or subcontractors in the course of, or incidental to, performing the Project or the use by the Commonwealth of reports provided by the Administering Organisation under this Agreement.

24.4 The indemnity referred to in clause 24.3 shall survive the expiration or termination of this Agreement.

25. Protection of Personal Information

25.1 The Administering Organisation agrees with respect to all activities related to or in connection with the performance of the Project or in connection with this Agreement:

(a) to comply with the Information Privacy Principles set out in section 14 of the *Privacy Act 1988*;

(b) not to transfer personal information held in connection with this Agreement outside Australia, or to allow parties outside Australia to have access to it, without the prior approval of the Commonwealth;

(c) to co-operate with any reasonable demands or inquiries made by the Privacy Commissioner or the CEO in relation to the management of personal information by the Administering Organisation, or breaches, or alleged breaches, of privacy;

(d) to ensure that any person who has an access level which would enable that person to obtain access to any personal information (as defined in the *Privacy Act 1988*) is made aware of, and undertakes in writing, to observe the Information Privacy Principles referred to in paragraph (a) above;

(e) to comply with any policy guidelines laid down by the Commonwealth or issued by the Privacy Commissioner from time to time relating to the handling of personal information;

(f) to comply with any reasonable direction of the CEO to observe any recommendation of the Privacy Commissioner relating to any acts or practices of the Administering Organisation that the Privacy Commissioner considers to be a breach of the obligations in paragraph (a) above;

(g) to comply with any reasonable direction of the CEO to provide the Privacy Commissioner access for the purpose of monitoring the Administering Organisation's compliance with this clause;

(h) to ensure that any record (as defined in the *Privacy Act 1988*) containing personal information provided to the Administering Organisation by the Commonwealth or any other person pursuant to this Agreement is, at the expiration or earlier termination of this Agreement, either returned to the ARC or deleted or destroyed in the presence of a person authorised by the ARC to oversee such deletion or destruction; and

(i) to the naming or other identification of the Administering Organisation in reports by the Privacy Commissioner.

25.2 The Administering Organisation must immediately notify the ARC if the Administering Organisation becomes aware of a breach of its obligations under clause 25.1.

25.3 This clause survives the expiration or earlier termination of this Agreement.

26. Confidentiality

26.1 Subject to clause 26.2, the ARC agrees not to disclose any Confidential Information of the Administering Organisation, without the Administering Organisation's consent.

26.2 The ARC will not be taken to have breached its obligations under clause 26.1 to the extent that the ARC discloses Confidential Information:

(a) to its officers, employees, agents, external professional advisers or contractors solely to comply with obligations, or to exercise rights, under this Agreement;

(b) to its internal management personnel solely to enable effective management or auditing of this Agreement or the National Competitive Grants Program or the Scheme;

(c) for a purpose directly related to the enforcement or investigation of a possible breach of any Commonwealth, State, Territory or local law;

(d) to the Minister, or in response to a demand by a House or a Committee of the

Commonwealth Parliament;

(e) within the ARC, the Department or another government agency or authority, where this serves the ARC's, the Department's or the Commonwealth's legitimate interests;

(f) as required or permitted by any other law, or an express provision of this Agreement, to be disclosed; or

(g) that is in the public domain other than due to a breach of this clause 26.

26.3 The Commonwealth warrants that, for a period of three years from the date of submission of the Final Report for the Project, it will consult with the Administering Organisation before any information which is contained in any reports related to the Project, and which the Administering Organisation has indicated is confidential and should not be disclosed, is disclosed to any person other than an officer, employee, agent or member of the ARC or the Minister. If disclosure is required to other persons, the Commonwealth will discuss the intended terms of disclosure with the Administering Organisation.

27. Acknowledgments, Publications and Publicity

27.1 Subject to commercial sensitivities and Intellectual Property considerations, the outcomes of Projects are expected to be communicated to the research community and, where appropriate and possible, to the community at large.

27.2 When, at any time during or after completion of a Project, the Administering Organisation or the researchers or any other party publishes, produces or is involved in promotional material, such as books, articles, television or radio programs, electronic media, newsletters or other literary or artistic works, which relate to the Project, the Administering Organisation must ensure (wherever possible) that the ARC's contribution and support of the Project is acknowledged in a prominent place and an appropriate form acceptable to the ARC. Similar efforts should be made when publicly speaking about a Project. Advice of acceptable forms of acknowledgement and use of the logo is provided on the ARC website.

27.3 This clause survives the expiration or earlier termination of this Agreement.

28. Administration of the Funding

28.1 The Administering Organisation must maintain reasonable, up-to-date and accurate records relating to the Funding in general, and the Projects conducted with the Funding in particular, to verify its compliance with this Agreement.

28.2 Records maintained under clause 28.1 must be retained by the Administering Organisation for each Project for a period of no less than seven years after whichever is the later of:

(a) the final payment of Funds by the ARC for the Project; or

(b) the final approved carryover of Funds for the Project.

29. Audit and Monitoring

29.1 The Administering Organisation is responsible for monitoring the expenditure of the Funding and certifying to the ARC that the Funding has been expended in accordance with this Agreement in the End of Year Report. If at any time, in the opinion of the Responsible Officer of the Administering Organisation, the Funding is not being expended in accordance with this Agreement, the Administering Organisation must take all action necessary to minimise further expenditure in relation to the Project and inform the ARC immediately.

29.2 The ARC may at any time conduct ad hoc on-site reviews to ensure that the terms of this Agreement are being, or were, met and that reports submitted to the ARC are an accurate statement of compliance by the Administering Organisation. Persons nominated by the ARC to conduct these reviews are to be given full access by the Administering

Organisation, if required, to all accounts, records, documents and premises in relation to the Funding and the administration of the Funds in general.

29.3 The Administering Organisation must:

(a) provide information to the Scheme Coordinator, or a person nominated by the Scheme Coordinator, as reasonably required by the Scheme Coordinator;

(b) comply with all reasonable requests, directions, or monitoring requirements received from the Scheme Coordinator; and

(c) cooperate with and assist the ARC in any review or other evaluation that the ARC undertakes.

30. Access to Premises and Records

30.1 The Administering Organisation must, at all reasonable times, give to the CEO or any person authorised in writing by the CEO:

(a) unhindered access to:

i. the Administering Organisation's employees;

ii. premises occupied by the Administering Organisation; and

iii. Material; and

(b) reasonable assistance to:

i. inspect the performance of any or all Projects;

ii. locate and inspect Material relevant to any Project or the Administering Organisation's compliance with this Agreement or the Scheme; and

iii. make copies of any such Material and remove those copies and use them for any purpose connected with this Agreement or the Scheme.

30.2 The access rights in clause 30.1 are subject to:

(a) any agreement to the contrary with a Partner Organisation which can be justified to the satisfaction of the ARC on the grounds of commercial sensitivity (including Intellectual Property considerations);

(b) the provision of reasonable prior notice by the ARC; and

(c) the Administering Organisation's reasonable security procedures.

30.3 If a matter is being investigated which, in the opinion of the ARC, or any person authorised in writing by the CEO, may involve an actual or apprehended breach of the law, clause 30.2 will not apply.

30.4 Upon receipt of reasonable written notice from the ARC, or any person authorised in writing by the CEO, the Administering Organisation must provide any information required by the Commonwealth for monitoring and evaluation purposes.

30.5 Nothing in clauses 30.1 to 30.4 inclusive affects the obligation of each party to continue to perform its obligations under this Agreement unless otherwise agreed between them.

30.6 The Auditor-General, or a delegate of the Auditor-General for the purpose of performing the Auditor-General's statutory functions, at reasonable times and on giving reasonable notice to the Administering Organisation, may:

(a) require the Administering Organisation to provide records and information which are directly related to this Agreement;

(b) have access to the premises of the Administering Organisation for the purposes of inspecting and copying documentation and records, however stored, in the custody or

under the control of the Administering Organisation which are directly related to this Agreement; and

(c) where relevant, inspect any Commonwealth Assets and Commonwealth Material held on the premises of the Administering Organisation.

30.7 This clause survives the expiration or earlier termination of this Agreement.

31. Reporting Requirements

31.1 The Administering Organisation must submit the following reports in accordance with this Agreement and the ARC Act, in the format required by the ARC, if specified.

31.2 End of Year Report

(a) The Administering Organisation must submit an End of Year Report by 31 March in the year following each full calendar year for which the Funding was awarded. The ARC will provide the Administering Organisation with a pro forma for this report.

(b) As part of the End of Year Report, the Responsible Officer must certify for each Project what the Partner Organisation Cash and In-Kind Contribution has been in relation to that Project. The Responsible Officer must also:

i. certify for each Project that the Partner Organisation Contribution has been provided in accordance with the written partner agreement entered into under clause 11, and that, to the best of her/his knowledge, the Partner Organisation does not intend to withdraw or reduce its contribution to the Project; or

ii. advise the ARC of any reduction or other anomalies in relation to the Partner Organisation Contribution for a Project.

(c) The End of Year Report will contain information on all expenditure for that year under the Project, on a Project-by-Project basis, including:

i. any unspent Funds to be recovered by the Commonwealth;

ii. any unspent Funds that the Administering Organisation is seeking to have carried over into the next year; and

iii. the reasons why the unspent Funds are required to be carried over.

(d) Under paragraph 58(1)(e) of the ARC Act, Funds provided by the Commonwealth to the Administering Organisation which are not spent during the year of the Funding Period to which those funds were allocated may be carried over if approved by the ARC. If the Administering Organisation wishes any Funds to be carried over from one year to the next year, the Administering Organisation must request this approval in the End of Year Report for the calendar year for which those Funds were initially paid.

(e) Where a carryover is requested for 75 per cent or more of the Funds allocated for a calendar year (excluding any Funds carried over from the previous calendar year), written justification must be provided.

(f) Funds may be carried over more than twelve months only <u>in exceptional circumstances</u> and subject to approval by the ARC. Separate written justification must be provided in this instance.

31.3 Progress Report

(a) Report by Exception. A Progress Report must only be submitted if significant issues are affecting the progress of the Project. The report must specify the actions being taken to address the issues. When required, the Administering Organisation must ensure that Project Leaders provide a Progress Report in respect of each affected ongoing Project by 31 March of each year, on a form which will be made available

on the ARC website. For the purposes of clarification, if a report by exception is required the initial Progress Report is due 31 March 2014.

(b) If the ARC is not satisfied with the progress of any Project, further payment of Funds will not be made until satisfactory progress has been made on the Project. If satisfactory progress is still not achieved within a reasonable period of time, the Funding may be terminated and all outstanding monies will be recovered by the ARC.

(c) Unsatisfactory progress on any Project may be noted against any further proposals under any ARC scheme submitted on behalf of the Chief Investigator and will be taken into account in the assessment of those proposals.

31.4 Final Report

(a) Unless otherwise approved by the ARC, the Administering Organisation must ensure that Final Reports are provided for each Project within twelve months of the final payment of Funds by the ARC for the Project or within twelve months of the final carryover of Funds approved by the ARC, whichever is the later. The form for this report will be made available by the ARC in RMS, with instructions on the ARC website. The ARC may review the outcomes against the objective(s) of the Project as stated in the Proposal or any approved revised budget, aims and research plan.

(b) The Final Report must justify why any publications from a Project have not been deposited in an open access institutional repository within twelve months of publication. The Final Report must outline how data arising from the Project have been made publicly accessible where appropriate.

(c) The ARC may also seek additional information about subsequent publications after submission of the Final Report.

(d) If a Final Report is considered by the ARC to be inadequate or is not submitted on time, the Administering Organisation of the Project may be contacted for further information. If the ARC is not satisfied with the outcomes of the Project, this may be noted against any further proposals under any ARC scheme submitted on behalf of any Chief Investigator on the Project and may be taken into account in the assessment of those proposals.

(e) Proposals submitted under any ARC scheme on behalf of any Chief Investigator on a Project for which the Final Report is outstanding may be deemed ineligible for approval of funding.

31.5 This clause survives the expiration or earlier termination of this Agreement.

32. Australian Research Integrity Committee

32.1 The Administering Organisation must provide to the Australian Research Integrity Committee (ARIC) upon request, any documentation and information concerning allegations of its mishandling of internal reviews into alleged cases of research misconduct, and/or non-compliance with the NHMRC/ARC/UA *Australian Code for the Responsible Conduct of Research* (2007).

33. Copyright in Proposals and Reports

33.1 Copyright in any Proposal and all reports provided under this Agreement will vest in the Administering Organisation at the time of creation but the Administering Organisation grants to the Commonwealth a permanent, irrevocable, royalty-free, non-exclusive licence to use and reproduce information contained in a Proposal or a report and publish it on a non-profit basis for any purpose related to:

(a) the evaluation and assessment of proposals;

(b) verifying the accuracy, consistency and adequacy of information contained in a Proposal, or otherwise provided to the ARC;

(c) the preparation and management of any funding agreement;

(d) the administration, auditing, management or evaluation of the National Competitive Grants Program or any funding scheme administered by the ARC;

(e) the sharing of information by the ARC within the ARC's organisation, or with another Commonwealth Department or agency, or Commonwealth Minister or parliamentary committee, where this serves the Commonwealth's legitimate interests; or

(f) where the use, reproduction or publication of the material is authorised or required by law.

33.2 If a Proposal or a report contains information belonging to a third party, the Administering Organisation must ensure that it has in place all necessary consents sufficient to allow the ARC to deal with the information or any report in accordance with this Agreement.

33.3 This clause survives the expiration or earlier termination of this Agreement.

34. Recovery of Unspent Funds or Overpayments of Funds

33.1 Any unspent Funds may be recovered by the Commonwealth under paragraph 58(1)(c) of the ARC Act. Any amount of Funding paid to the Administering Organisation which exceeds the amount of financial assistance that is properly payable to it may be recovered under paragraph 58(1)(d) of the ARC Act.

34.2 The Commonwealth may offset the unspent or overpaid Funds against the total of any further Funds payable to the Administering Organisation.

34.3 This clause survives the expiration or earlier termination of this Agreement.

35. Indemnity

35.1 The Administering Organisation, if it is not a Commonwealth Entity which is contracting on behalf of the Commonwealth, will indemnify the Commonwealth, its officers, employees and agents against any liability, loss, damage, costs and expenses arising from any claim, suit, demand, action or proceeding by any person, where such loss or liability was caused by a wilful, unlawful or negligent act or omission of the Administering Organisation, its employees, agents or subcontractors in connection with this Agreement.

35.2 The Administering Organisation's liability to indemnify the Commonwealth under clause 35.1 shall be reduced proportionally to the extent that any act or omission of the Commonwealth or its employees or agents contributed to the loss or liability.

35.3 The indemnity referred to above shall survive the expiration or termination of this Agreement.

36. Insurance

36.1 The Administering Organisation must effect and maintain adequate insurance or similar coverage to cover any liability arising as a result of its participation in *Linkage Projects* Projects and, if requested, provide the Commonwealth with a copy of the relevant policies or when appropriate Certificate of Currency. The Administering Organisation will be responsible for effecting all insurances required under worker's compensation legislation and for taking all other action required as an employer.

37. Dispute Resolution

37.1 The Parties agree not to commence any legal proceedings in respect of any dispute arising

under this Agreement until the procedure provided by this clause 37 has been followed.

37.2 The Parties agree that the following procedure will be used in an expeditious way to resolve a dispute:

(a) the Party claiming there is a dispute will notify the other Party in writing, setting out the nature of the dispute;

(b) the Parties will try to resolve the dispute through direct negotiation, including by referring the matter to persons who have authority to intervene and direct some form of resolution;

(c) the Parties have ten business days from the date of the notice to reach a resolution or to agree that the dispute is to be submitted to mediation or some alternative dispute resolution procedure

(d) if:

i. there is no resolution of the dispute;

ii. there is no agreement on submission of the dispute to mediation or some alternative dispute resolution procedure; or

iii. there is a submission to mediation or some other form of alternative dispute resolution procedure, but there is no resolution within 30 business days of the submission, or such extended time as the Parties may agree in writing before the expiration of the 30 business days;

then, either Party may commence legal proceedings.

37.3 This clause 37 does not apply to action that the Commonwealth takes, or purports to take, under clause 5, 34 or 38 or where a Party commences legal proceedings for urgent interlocutory relief. Clause 5 relates to payment of Funds, clause 34 relates to recovery of unspent Funds or overpayments of Funds, clause 38 relates to termination of the Agreement.

37.4 Despite the existence of the dispute, the Administering Organisation must (unless requested in writing by the Commonwealth not to do so) continue to perform its obligations under this Agreement.

38. Termination of the Agreement

38.1 The ARC may immediately terminate Funding for a Project by notice in writing to the Administering Organisation if:

(a) the Administering Organisation commits any breach of this Agreement which the Commonwealth considers is not capable of remedy;

(b) in the opinion of the ARC, progress on the Project is not satisfactory;

(c) the ARC reasonably believes that it has received inaccurate, incomplete or misleading information in relation to that Project, including in the Proposal or in any report provided under this Agreement;

(d) the ARC reasonably believes there is fraud or misleading or deceptive conduct on the part of the Administering Organisation or any Specified Personnel;

(e) the Administering Organisation fails to comply with any additional ARC requirement or condition notified by the ARC under clause 3.2;

(f) the ARC receives notice that work on the Project will cease, or has ceased;

(g) the Administering Organisation is unable to continue its role for any reason;

(h) the Commonwealth terminates its commitment in the event of a change of government policy or other related government requirements.

to provide the Administering Organisation with an acknowledgment that the officer, employee, agent or subcontractor is aware of the provisions of the section.

Notes: Administering Organisations should note also that they may be subject to the provisions and applications of the *Competition and Consumer Act 2010* and the *Archives Act 1983*. More information about the *Charter of United Nations Act 1945* and the *Charter of United Nations (Terrorism and Dealing with Assets) Regulations 2002* is available at www.dfat.gov.au/icat/UNSC_financial_sanctions.html.

39.4 The Administering Organisation must ensure that in performing the obligations under, and arising from this Agreement, all necessary precautions for the health and safety of all persons have been identified and implemented.

39.5 The Administering Organisation must ensure that all officers, employees, agents and subcontractors in performing the obligations under and arising from this Agreement comply with any security and safety requirements of which they are or should be reasonably aware.

40. Liaison

40.1 Researchers should direct all queries regarding ARC Funding to their Administering Organisation's Research Office in the first instance. All communications from the Administering Organisation to the ARC or the Minister, relating to the Funding should be made through the Responsible Officer of the Administering Organisation and should be directed to the Scheme Coordinator at the following address:

Scheme Coordinator
Linkage Projects Phone: 02 6287 6600
Australian Research Council Fax: 02 6287 6638

GPO Box 2702
CANBERRA ACT 2601
Email: ARC-LinkageProjects@arc.gov.au

Post-award enquiries and report submission
Email: ARC-postaward@arc.gov.au

41. Applicable Law

41.1 This Agreement will be governed by and interpreted in accordance with the laws of the Australian Capital Territory and the Parties submit to the non-exclusive jurisdiction of the courts of that Territory.

SCHEDULE B

Research special conditions

B1. Importation of experimental organisms: The Administering Organisation must ensure that, before experimental organisms are imported into Australia for the purposes of a Project, the Administering Organisation or the Chief Investigator of the Project must obtain agreement in principle for the importation from the appropriate Commonwealth and State authorities.

B2. Research involving humans or other animals: If any Project conducted by the Administering Organisation involves research on or involving humans or other animals, the Administering Organisation must ensure that the codes adopted for these purposes by the National Health and Medical Research Council, Australian Research Council and Universities Australia are complied with. The Project may not commence without clearance from the Administering Organisation's Ethics or Biosafety Committee (or equivalent) and from any other relevant authority.

B3. Deposition of biological materials: Any biological material accumulated during the course of a Project shall be transferred to an Australian body with statutory responsibility for control of such material. If no such body is available to take control of the biological material then the Administering Organisation must dispose of the material in accordance with the Administering Organisation's established safeguards.

B4. Genetic manipulation: If a Project involves the use of gene technology (as defined in the *Gene Technology Act 2000*), then before the proposed research commences, the Administering Organisation must ensure that the research has been approved in writing by the relevant Biosafety and/or Ethics Committees (or equivalent) of the Administering Organisation. The Administering Organisation must retain all certificates relating to the above and will provide evidence to the Scheme Coordinator if required to do so. The Administering Organisation must be accredited with the Office of the Gene Technology Regulator.

B5. Ionising radiation: If a Project involves the use of ionising radiation, the Administering Organisation shall ensure that any personnel performing procedures involving ionising radiation are appropriately trained and hold a relevant current licence from the appropriate State authority. The Administering Organisation must retain all such licences and shall provide them to the Scheme Coordinator if required to do so.

B6. Social science data sets: Any digital data arising from a Project involving research relating to the social sciences should be lodged with the Australian Social Science Data Archive (ASSDA) for secondary use by other investigators. This should normally be done within two years of the conclusion of any fieldwork relating to the Project research. If a Chief Investigator is not intending to do so within the two-year period, he/she should include the reasons in the Project's Final Report.

/

SCHEDULE C

Evaluation of the adequacy of Partner Organisation cash and in-kind contributions for the Linkage Projects Scheme

This Schedule is to be used in determining the value of Partner Organisation Contributions that are required to be provided by Partner Organisations to the Administering Organisation to at least match dollar-for-dollar the financial assistance to be provided by the Commonwealth for Projects within the *Linkage Projects* Scheme.

C1. Underlying principles and practical considerations

C1.1 The primary objectives in examining the Partner Organisation Contribution are to ensure that:

(a) the available support is adequate for the successful completion of the Project;

(b) the total eligible Partner Organisation Contribution matches at least dollar-for-dollar the financial assistance provided by the Commonwealth over the life of the Project. (Section 6.3 of the Funding Rules describes the financial commitment requirements of Partner Organisations); and

(c) the Partner Organisation Contribution is in accordance with the budget, aims and research plan contained in the Proposal or an approved revised budget, aims and research plan.

C1.2 The Partner Organisation Contribution requirements specified in clause 11.5 of this Agreement must be met.

C1.3 In-kind contributions that are shown to be essential and central to the conduct of the Project are given full recognition in evaluating the eligible Partner Organisation Contribution. The onus is on the Administering Organisation to establish the merit of the case for recognition of the level and extent of the in-kind contributions. As a general rule, the value of in-kind contributions should reflect current internal and non-commercial rates.

C1.4 If the amount of total Commonwealth Funding for a Project varies from the amount sought in the Proposal for the Project, the Project Leader must discuss the matter with the Partner Organisation. If the Partner Organisation agrees that the research Project is viable within the parameters of the varied amount of Commonwealth funding, pro-rata adjustments may be made to the Proposal budget in accordance with clause 11.

C1.5 The Project Leader is responsible for providing the Research Office of the Administering Organisation with evidence of any such Partner Organisation agreement for Funding acquittal and reporting purposes.

C1.6 In-kind contributions to a Project may include, but are not restricted to, scientific liaison and management, direct technical support, or unique access to reagents, data, processors or equipment.

C1.7 Corporate membership or subscription fees in industrial consortia do not qualify as Partner Organisation Contributions but the allocation of designated research funds, together with the identification of the linkages between the member and the Project, do qualify.

IN WITNESS WHEREOF the parties have agreed to this Agreement on the date first above written.

SIGNED for and on behalf of
THE COMMONWEALTH OF AUSTRALIA

by Dr Laura Dan
insert name of signatory above

the Chief Program Officer
insert signatory's title above
of the Australian Research Council

In the Presence of:

Amber Hesketh
insert name of witness above

signatory to sign above

witness to sign above

SIGNED for and on behalf of
The University of Newcastle

by PROF DEBORAH HODGSON
insert name of signatory above

the PRO VICE CHANCELLOR FOR RESEARCH AND INNOVATION
insert signatory's title above
of the said Administering Organisation who, by signing, certifies that he/she has the authority so to sign

In the Presence of:

TIMOTHY DGAN
insert name of witness above

signatory to sign above

witness to sign above
