# Supplementary material for: A randomised controlled trial of an intervention to increase the implementation of a healthy canteen policy in Australian primary schools: study protocol
Source: Implement Sci. 2014 Oct 11;9:147. doi: 10.1186/s13012-014-0147-3 (PMC4197283; doi:10.1186/s13012-014-0147-3)
Supplement: Additional file 3: — Collaborative research agreement between The university of Newcastle and Hunter New England Local Health District. [file 13012_2014_147_MOESM3_ESM.pdf]

THE UNIVERSITY OF
NEWCASTLE
AUSTRALIA

Australian Research Council
Linkage Projects Scheme 2013

COLLABORATIVE RESEARCH AGREEMENT

between

THE UNIVERSITY OF NEWCASTLE
ABN 15 736 576 735

and

HUNTER NEW ENGLAND LOCAL HEALTH DISTRICT
ABN 63 598 010 203

in relation to the project:

"Moving from policy to practice: A randomised trial of an implementation intervention to facilitate the adoption of a statewide healthy canteen policy"

THIS AGREEMENT is made on ..11..February.. 2014

BETWEEN

The University of Newcastle ABN 15 736 576 735, a body corporate constituted pursuant to the *University of Newcastle Act 1989* (NSW) of University Drive, Callaghan, NSW 2308 **(UON)**

AND

Each Eligible Organisation and Partner Organisation specified in Schedule 1 (the **Collaborating Organisations**).

BACKGROUND

A. UON with the assistance of the Collaborating Organisations lodged the Proposal for the Project under the ARC 'Linkage Projects Scheme'.

B. UON together with the Collaborating Organisations was successful and was awarded the ARC Funds.

C. UON has entered into the Funding Agreement with the ARC in relation to the ARC Funds and the conduct of the Project.

D. This Agreement sets out the terms and conditions under which UON and the Collaborating Organisations have agreed to undertake the Project.

OPERATIVE PROVISIONS

1. Definitions and Interpretation

1.1 Definitions

In interpreting this Agreement the following words and expressions have the following meanings:

"Administering Organisation" has the meaning given in the Funding Agreement.

"ARC" means the Australian Research Council as established pursuant to the *Australian Research Council Act 2001* (Cth).

"ARC Funds" means the funds provided by the ARC to UON under the Funding Agreement.

"Background Intellectual Property" means a Party's Intellectual Property which existed prior to the commencement of the Project which that Party makes available for the carrying out of the Project or which a Party develops

independently of the Project and includes any Background Intellectual Property listed in Item 8 of Schedule 1.

"Chief Investigator" means the person(s) named as such in the Proposal and listed in Item 4 of Schedule 1.

"Collaborator Contributions" means the Collaborator Cash Contribution and the Collaborator In-Kind Contribution.

"Collaborator Cash Contribution" means the cash contribution to be made to the Project by a Collaborating Organisation in Schedule 2.

"Collaborator In-Kind Contribution" means the in-kind contribution to be made to the Project by a Collaborating Organisation in Schedule 2.

"Collaborating Organisations" means each Eligible Organisation and Partner Organisation listed in Schedule 1.

"Collaborator's Premises" means the offices, workshops, laboratories, factories and other buildings or space occupied or controlled by a Collaborating Organisation required or utilised in relation to the conduct of the Project.

"Commencement Date" means the day on which the last Party executes this Agreement or such other date agreed in writing between the Parties provided that such date is prior to the Project Sunset Date.

"Commercialise" means, in relation to the Project Intellectual Property, to manufacture, sell, hire, or otherwise Exploit a product, process or service, incorporating or derived from the Project Intellectual Property (whether or not for profit), or to licence any third party to do any of those things and "Commercialisation" is to be similarly construed.

"Confidential Information" means all know-how, financial information and other commercially valuable or sensitive information in whatever form, including inventions (whether or not reduced to practice), trade secrets, formulae, graphs, drawings, biological materials, samples, devices, models and any other materials or information of whatever description which a Party regards as confidential, proprietary or of a commercially sensitive nature that may be in the possession of a Party's employees or management.

"Conflict of Interest" has the meaning given in the Funding Agreement.

"Eligible Organisation" has the meaning given in the Funding Agreement.

"Exploit" means:

i) in relation to an Intellectual Property right, the exercise of the rights exclusively granted to the holders of such Intellectual Property right by the laws of the jurisdiction in which the Intellectual Property right subsists;

ii) in relation to a product, kit, apparatus, substance, documentation or information resource (or any part of such materials), to make, distribute, market, sell, hire out, lease, supply, or otherwise dispose of it; and

iii) in relation to a method or process, to use the method or process or to make, distribute, market, sell, hire out, lease, supply, or otherwise dispose of a product, kit or apparatus the use of which is proposed or intended to involve the exercise of the method or process.

"Force Majeure" means an act of God, fire, lightning, explosion, flood, subsidence, insurrection or civil disorder or military operation, government or quasi-government restraint, expropriation, prohibition, intervention, direction or embargo, inability or delay in obtaining governmental or quasi-governmental approvals, consents, permits, licences or authorities, strikes, lock-outs or other industrial disputes of any kind and, any other cause whether similar or not to the foregoing, outside of the affected Party's control.

"Funding Agreement" means the agreement between UON and the Commonwealth of Australia, as represented by the ARC, pursuant to which the Commonwealth provides the ARC Funds to UON, a copy of which is annexed as Annexure B.

"Funding Rules" means the ARC *'Linkage Projects Funding Rules for funding commencing in 2013'* as varied or replaced from time to time.

"GST Law" means *A New Tax System (Goods and Services Tax) Act 1999* (Cth).

"Insolvency Event" means circumstances in which a Party is:

i) unable to pay its debts as and when they fall due and payable, makes or commences negotiations with a view to making a general re-scheduling of its indebtedness, a general assignment, scheme of arrangement or composition with its creditors;

ii) takes any corporate action or any steps are taken or legal proceedings are started for:

   a) its winding-up, dissolution, liquidation (voluntary or otherwise), or re-organisation, other than to reconstruct or amalgamate while solvent on terms approved by the other Party (which approval will not be unreasonably withheld); or

   b) the appointment of a controller, receiver, administrator, official manager, trustee or similar officer of it or of any of its revenues and assets (whether voluntary or otherwise); or

iii) seeks protection or is granted protection from its creditors, under any applicable legislation.

"Intellectual Property" means statutory and other proprietary rights in respect of copyright and neighbouring rights; all rights in relation to inventions, patents,

plant varieties, registered and unregistered trademarks, registered and unregistered designs, circuit layouts and confidential information; and all other rights with respect to Intellectual Property as defined in Article 2 of the July 1967 Convention Establishing the World Intellectual Property Organisation (as amended from time to time).

"Minister" has the meaning given in the Funding Agreement.

"Moral Rights" means all present and future rights of integrity of authorship, rights of attribution of authorship, rights not to have authorship falsely attributed and rights of a similar nature conferred by statute anywhere in the world.

"Parties" means the parties to this Agreement and **"Party"** means any one of them.

"Partner Investigator" means the person(s) named as such in the Proposal and listed in Item 5 of Schedule 1.

"Partner Organisation" has the meaning given in the Funding Agreement.

"Post Graduate Research Student" means a higher degree by research candidate who is expected to work on the Project.

"Project" means the program of research described in Item 1 of Schedule 1.

"Project Assets" means the assets (whether tangible or not) purchased as part of the Project with funds provided by UoN, a Collaborating Organisation or the ARC Funds but does not include any Project Intellectual Property purchased or associated with such assets.

"Project Control Group" means the consultative forum for the conduct of the Project referred to in clause 5.

"Project Cost" means the cost and proposed funding summary for the Project contained in the Proposal.

"Project Description" means the description of the Project contained in the Proposal.

"Project Intellectual Property" means the Intellectual Property rights which are created, developed or conceived by any of the Parties during the course of or as a result of the carrying out of, or association with, the Project.

"Project Sunset Date" means the last day for the commencement of the Project in the Funding Agreement and listed in Item 7 of Schedule 1.

"Proposal" means the *'ARC Linkage-Projects Proposal for Funding Commencing in 2013'* LP130101008 regarding the Project, a copy of which is annexed as Annexure A.

"Research Plan" means the plan for the conduct of the Project determined by the Project Control Group.

"Research Purposes" means the internal and non-commercial use of Project Intellectual Property for teaching, research and scholastic purposes but does not include the right to Commercialise, assign or sub-licence Project Intellectual Property.

"Schedule" means a Schedule to this Agreement.

"Specified Acts" means in relation to Moral Rights any of the following classes or types of acts or omissions by or on behalf of UON:

i) using, reproducing, modifying, adapting, publishing, performing broadcasting, communicating, Commercialisation or the exploitation of all or any part of the Project Intellectual Property and/or Background Intellectual Property, with or without attribution of authorship;

ii) supplementing the Project Intellectual Property and/or Background Intellectual Property with any other content or material; and

iii) using the Project Intellectual Property and/or Background Intellectual Property in a different context to that originally envisaged.

"Term" means the period during which the Project is expected to be undertaken as set out in Item 6 of Schedule 1.

"UON Cash Contribution" means the in-kind contribution to be made to the Project by UoN listed in Schedule 2.

"UON Contributions" means the UON Cash Contribution and UON In-Kind Contribution.

"UON In-Kind Contribution" means the in-kind contribution to be made to the Project by UoN listed in Schedule 2.

1.2 Interpretation

In this Agreement, unless a contrary intention appears from the context:

a. Words importing the singular only also include the plural and vice versa and words denoting a given gender include both genders.

b. Words such as 'includes' or 'including' are not words of limitation and do not impose any limitation on the construction of general language followed by specific examples.

c. Clause headings and notes in square brackets are not part of the conditions of the Agreement and are not to be taken into consideration in the interpretation or construction thereof, except for the purpose of rectifying any erroneous cross reference.

d. All monetary amounts referred to in this Agreement are in Australian currency.

e. All references to clauses are references to clauses in this Agreement, unless the contrary is apparent from the context.

f. A reference to legislation (including subordinate legislation) is a reference to that legislation as amended, consolidated, re-enacted or replaced, and includes any subordinate legislation issued under it.

g. A reference to a person or body which has ceased to exist or has been reconstituted, amalgamated or merged, or the functions of which have become exercisable by another person or body, is to be taken as a reference to the person or body established or constituted in its place or by which its functions have become exercisable.

2 Funding Agreement

2.1 Each Collaborating Organisation warrants that:

a. it is aware UON has entered into, and is the Administering Organisation under, the Funding Agreement;

b. it has read and understood the terms of the Funding Agreement and has provided a copy of the Funding Agreement to each of its Chief Investigators or Partner Investigators (as the case may be);

c. it will comply with and abide by the terms of the Funding Agreement including any future requirements, conditions and restrictions imposed by the Minister or ARC as if the Collaborating Organisation were a party to the Funding Agreement provided that:

   i. reference to the Administering Organisation will be interpreted as being a reference to the Collaborating Organisation;

   ii. reference to ARC or the Commonwealth will be interpreted as being a reference to UON; and

   iii. nothing in this Agreement confers upon the Collaborating Organisation the right to exercise any discretion or right of UON under the Funding Agreement;

d. it will do all things incidental or reasonably necessary to give effect to the Funding Agreement;

e. it will not impede or prevent compliance or do or omit to do anything that will cause or contribute to UON not being able to comply with its obligations under the Funding Agreement; and

f. it must provide all assistance UON reasonably requires to allow UON to discharge its obligations under the Funding Agreement including allowing unhindered access to any Collaborator's Premises or to inspect its records relating to the ARC Funds and the Project where such access or inspection

is reasonably required (as determined by UON) to allow UON to comply with its obligations under the Funding Agreement.

2.2 UON will use its best endeavours to:

a. meet its obligations under the Funding Agreement and will notify the Collaborating Organisations if the Funding Agreement is varied or terminated for any reason; and

b. provide the Collaborating Organisations with a copy of any annual report which it provides to the ARC in compliance with the Funding Agreement and any required progress reports (which may not necessarily be in writing) under the Funding Agreement.

2.3 The provisions of the Funding Agreement are to be read in conjunction with this Agreement. Where there is any inconsistency between the provisions of this Agreement and those of the Funding Agreement, the latter will prevail.

2.4 Nothing in this Agreement will reduce or otherwise affect the rights, entitlements and obligations of UON under the Funding Agreement or is intended to impede or prevent UON from complying with any of its obligations under the Funding Agreement.

3 ARC Funds

3.1 Subject to the Collaborating Organisations complying with their obligations under this Agreement and compliance with the Funding Agreement, UON will, if applicable, distribute the ARC Funds to the Collaborating Organisations in accordance with the Funding Agreement and the Proposal.

3.2 The Collaborating Organisations acknowledge and agree that:

a. the amount of the ARC Funds distributed (if any) may be less than that shown in the Proposal; and

b. the distribution of the ARC Funds is subject at all times to UON receiving the ARC Funds in full from ARC for the Project.

3.3 Each Collaborating Organisation acknowledges and agrees that, to the extent it receives any of the ARC Funds, it will ensure that the ARC Funds are strictly spent:

a. in accordance with the Project Cost;

b. otherwise in accordance with the Proposal; and

c. in accordance with the Funding Rules and Funding Agreement including the obligation not to expend ARC Funds for any purpose specifically excluded under the Funding Rules or Funding Agreement.

3.4 Any Project expenditure incurred by a Collaborating Organisation additional to the approved amount for the Project (if any) specified in this Agreement is the

responsibility of the Collaborating Organisation and there will be no entitlement to further ARC Funds.

3.5 If ARC or the Minister subsequently varies, withholds or withdraws the ARC Funds:

a. UON will notify the Collaborating Organisations;

b. the Parties must meet to determine in good faith whether the Project will proceed (and any required changes to respective contributions); and

c. UON retains the absolute right to terminate this Agreement upon the provision of written notice to the Collaborating Organisations.

3.6 Each Collaborating Organisation must immediately upon demand by UON repay the ARC Funds (or such proportion as required by UON) provided that UON will only exercise its rights under this clause if the ARC has required the repayment of all or part of the ARC Funds under the Funding Agreement.

4 Term of the Project

4.1 The Project is to commence on the Commencement Date and will continue until the expiry of the Term, unless the Project is extended by agreement between the Parties or terminated lawfully by a Party in accordance with this Agreement.

4.2 Upon termination of the Project for any reason other than breach of this Agreement by UON or completion of the Project, the Parties agree to share the cost of winding up the Project. The share to be contributed by the Parties, including a share of the cost of any Post Graduate Research Student stipend for the Term, will not exceed the value of the Collaborator Cash Contribution or UON Cash Contribution as the case may be.

5 Conduct of the Project

5.1 The Collaborating Organisations agree that UoN has primary responsibility for the conduct of the Project under the Funding Agreement.

5.2 The Parties acknowledge their respective roles for the Project set out in the Proposal and the Parties agree that they will use their best endeavours with due care and skill to ensure that the Project is:

a. conducted in accordance with the Funding Agreement (including any special conditions therein) and with any other requirements or conditions imposed by the ARC;

b. conducted in accordance with the Project Description and otherwise in accordance with the Proposal;

c. conducted in accordance with research best practice and with all applicable laws; and

d. commenced by no later than the Project Sunset Date.

5.3 As soon as reasonably practicable after the Commencement Date (but no later than 2 months after the Commencement Date), the Parties will each nominate a representative to participate on the Project Control Group. Unless approved by UON (such approval not to be unreasonably withheld), the representative must be a Chief Investigator or Partner Investigator (as the case may be).

5.4 The Project Control Group:

a. as soon as reasonably practicable (but no later than 3 months after the Commencement Date) must collaborate in good faith to determine the Research Plan;

b. must regularly review the progress of the Project and keep the Parties informed;

c. must keep records and minutes of any meetings and discussions in accordance with research best practice;

d. must use its best endeavours to ensure the Project proceeds in accordance with the Research Plan and the requirements listed in clause 5.2 and must immediately notify the Parties of any deviation or delay; and

e. is to act as a forum for consultation regarding the Project provided that the Project Control Group itself has no authority to bind a Party or exercise any discretion or right conferred upon a Party under this Agreement.

6 Collaborating Organisations - Project Obligations

6.1 Each Collaborating Organisation:

a. warrants that it will meet its obligations to the Project as set out in the Proposal diligently and competently and will notify UON immediately upon the happening of any event or occurrence that adversely affects the Collaborating Organisation's ability to meet its obligations to the Project;

b. warrants that it will obtain any required ethical clearances required under the Funding Agreement or otherwise required for the Project;

c. warrants that it will use its best endeavours to collaborate and work with UON in carrying out the Project including providing regular progress reports and other information reasonably required by UON from time to time to allow UON to comply with its obligations under the Funding Agreement including its reporting obligations to ARC;

d. warrants that it will provide the resources to undertake the Project as set out in the Proposal and warrants that any equipment required for the Project:

i. will not be encumbered or otherwise disposed of and not replaced in a manner that adversely affects the conduct of the Project;

ii. will be made available as and when reasonably required and is in good working order and condition and regularly maintained in accordance with the suppliers/manufacturer's recommendations; and

iii. is safe for its intended use and the Collaborating Organisation will provide copies of any risk assessment and safe operating procedure (or similar);

e. warrants that it will take, and ensure that its Chief Investigators or Partner Investigators (as the case may be) take, reasonable care of, and safely store any data or specimens or samples collected during, or resulting from, the conduct of the Project;

f. warrants that it will maintain reasonable, up-to-date and accurate records relating to the expenditure of ARC Funds and the conduct of the Project in accordance with research best practice;

g. warrants that it will comply with clause 7.20 of the Funding Agreement in respect of any Post Graduate Research Student stipend;

h. warrants that all information provided by the Collaborating Organisation for inclusion in the Proposal was complete, accurate and not misleading to the best of its knowledge and belief;

i. must use its best endeavours to ensure that any information provided to UON in relation to the Project is complete, accurate and not misleading;

j. warrants that its relationship with UON, the Chief Investigators and Partner Investigators complies with the requirements specified in the Funding Rules, and where it is a Partner Organisation that:

   i. the Collaborating Organisation's relationship with the Chief Investigators for the Project does not generate or represent a Conflict of Interest; and

   ii. the Collaborating Organisation satisfies the requirements for a Partner Organisation in the Funding Rules (if applicable); and

   iii. the Collaborating Organisation will make an appropriate contribution of time and operating costs towards the Project in relation to the participation of Partner Investigators in the Project,

   and the Collaborating Organisation indemnifies UON for any loss, liability or expense incurred or suffered by UON as a result of any breach of the Funding Agreement caused or contributed to by UON's reliance on the warranties in this clause 6.1j; and

k. must immediately notify UON of any actual or potential Conflict of Interest which has or may have the potential to influence or appear to influence the research and or activities, publications and media reports or requests for funding related to the Project.

6.2 The Collaborating Organisations will respect the position of any Postgraduate Research Student working on the Project acknowledging that they are subject to the supervision, leave and other arrangements of UON.

6.3 The Collaborating Organisations will permit employees, students or representatives of UON to visit the Collaborator's Premises at all reasonable times for the purposes of the Project.

7 Collaborating Organisations – Project Contributions

7.1 The Collaborating Organisations:

a. will provide UON with the Collaborator Contributions in the amount and at the times shown in Schedule 2; and

b. warrant that the Collaborator Contributions are made in accordance with and satisfy the requirements of the Funding Rules.

7.2 Notwithstanding the provisions of this or any other contract between the Parties, the Collaborating Organisations will not under any circumstances deduct or setoff any amount payable by UON from or against the Collaborator Contributions due to UON under this Agreement.

7.3 Any Collaborator Cash Contribution not paid on the due date immediately becomes a debt due and payable to UON without the need for further demand or proof of the debt.

7.4 UON will use Collaborator Contributions for the Project and for no other purpose.

8 UON – Project Contributions

8.1 UON will provide the UON Contribution to the Project (including the ARC Funds) in the amounts and at the times set out in Schedule 2.

9 Chief and Partner Investigators

9.1 The Parties agree that the Chief Investigators appointed by UON and nominated in Schedule 1, have authority to manage the day to day conduct of the Project in any manner that is not inconsistent with the Proposal and the requirements of this Agreement.

9.2 Each Collaborating Organisation warrants that each of its Chief Investigators or Partner Investigators (as the case may be):

a. meets the criteria for a Chief Investigator or Partner Investigator (as the case may be) specified in the Funding Rules and will continue to do so for the full duration of their involvement in the Project and must immediately notify UON of any change to this status;

b. will have direct responsibility for the strategic decisions and the communication of results for the Project as required under the Funding Rules and Funding Agreement;

c. has access to adequate resources and 'basic facilities' (as defined in clause 7.4 of the Funding Agreement);

d. has adequate time and the assistance reasonably required to commit to the Project as outlined in the Proposal including any required 'FTE' equivalent contribution identified in the Proposal;

e. will conduct the Project in a diligent and competent manner in accordance with the Proposal;

f. will comply with the Funding Agreement;

g. is eligible to perform the Project and that the Collaborating Organisation has made proper enquiries to ascertain this eligibility; and

h. will comply with the obligation to assess new proposals for ARC funding as required by ARC as set out in the Funding Agreement.

9.3 Each Collaborating Organisation must immediately notify UON if any of its Chief Investigators or Partner Investigators (as the case may be) is not able to undertake or continue to undertake the Project and must provide all assistance reasonably required by UON to :

a. comply with its obligations under the Funding Agreement including under clause 16 of the Funding Agreement; and

b. to identify a suitably qualified and experienced replacement.

10 Confidential Information

10.1 The ownership of Confidential Information which exists prior to the commencement of the Project will not be altered or transferred merely by virtue of its use in the Project.

10.2 All Confidential Information submitted by one Party to another Party, whether existing prior to the commencement of the Project or created during the course of the Project, will be kept confidential and will not be disclosed to any third party without the prior written consent of the disclosing Party, such consent will not be unreasonably withheld.

10.3 Notwithstanding clause 10.2, a Party is not obliged to keep the following information confidential:

a. information which is lawfully in the public domain prior to its disclosure to a Party by another Party;

b. information which subsequently enters the public domain otherwise than as a result of an unauthorised disclosure;

c. information which is or becomes available to the recipient Party from a third party lawfully in possession thereof and who has the lawful power to disclose such information to the recipient Party on a non-confidential basis; and

d. information which is rightfully known by the recipient Party (as shown by its written record) prior to the date of disclosure to it hereunder.

10.4 A Party that relies on one or more of the exceptions set out in clause 10.3 in all instances bears the onus of proof that the exception applies in the particular circumstance.

10.5 On termination of this Agreement, or upon the written request of UON, the Collaborating Organisations will return to UON all documents and copies of documents containing Confidential Information received or obtained from UON in connection with the Project.

10.6 Each Party will assume responsibility and liability for the actions of its employees, contractors, subcontractors, agents and representatives who have access to the Confidential Information of the other Party from time to time and will ensure that they are aware of and have agreed to be strictly bound by the confidentiality obligations created under this Agreement.

10.7 Any Confidential Information communicated to a Party hereunder may be disclosed by that Party to a sub-licensee properly appointed in accordance with this Agreement if the information disclosed is essential to the Project and the licence and the disclosure is limited to officers or employees of the sub-licensee who cannot properly fulfill their duties without such disclosure and who undertake in writing to keep such information confidential and to abide in all respects with the obligations contained in this Agreement so far as they relate to Confidential Information.

11 Background Intellectual Property

11.1 Subject to this Agreement, ownership of Background Intellectual Property will not be altered or transferred merely by virtue of its use in the Project.

11.2 Each Party grants to each other an irrevocable, non-exclusive, non-transferable, royalty-free licence to use its Background Intellectual Property during the Term to the extent necessary for that party to fulfill its obligations under this Agreement in relation to the conduct of the Project and for no other purpose provided that:

a. Background Intellectual Property of a Party must be kept confidential and otherwise treated as 'Confidential Information' in accordance with clause 10; and

b. each Party must use its best endeavours to protect the Background Intellectual Property including in relation to unauthorised access or use.

11.3 For the avoidance of doubt, the licence granted pursuant to clause 11.2 does not confer a right to sub-licence or to Commercialise the Background Intellectual Property.

11.4 Each Party warrants that to the best of its knowledge and belief the grant of the licence pursuant to clause 11.2 does not infringe (and has not been or is not alleged to infringe) the Intellectual Property rights or Moral Rights of any third party and must notify the other Parties of any alleged infringement from time to time.

12 Project Intellectual Property

12.1 Project Intellectual Property created or developed in the course of the Project will vest in, and be owned by, UON.

12.2 The Collaborating Organisations agree to do all such things and take any such action necessary to give effect to UON's ownership of Project Intellectual Property referred to in clause 12.1 including:

a. executing all documentation; and

b. performing all such acts,

required to transfer or assign ownership to UON provided that nothing in this clause requires a Collaborating Organisation to transfer or assign ownership of its Background Intellectual Property.

12.3 The Parties will consult on the best methods to protect any Project Intellectual Property having regard to UON's rights under this Agreement and the obligations of UON under the Funding Agreement to ensure that Project Intellectual Property is protected on reasonable commercial terms.

12.4 To the extent that the Background Intellectual Property of a Collaborating Organisation:

a. forms part of Project Intellectual Property; or

b. is not severable from Project Intellectual Property,

so as to not allow the effective use of, including to Commercialise, Project Intellectual Property by UON without the Background Intellectual Property, then each Collaborating Organisation grants to UON an irrevocable, non-exclusive, world-wide, royalty-free licence to use its Background Intellectual Property for the purposes listed in clause 12.5.

12.5 UON may use the Background Intellectual Property of a Collaborating Organisation for the following permitted uses:

a. Research Purposes;

b. subject to clause 14, to Commercialise Project Intellectual Property; and

c. for the purposes of granting the licence to other Collaborating Organisations referred to in clause 12.7.

12.6 Each Collaborating Organisation warrants that to the best of its knowledge and belief the grant of the licence pursuant to clause 12.4 does not infringe (and has not been or is not alleged to infringe) the Intellectual Property rights or Moral Rights of any third party and must notify UON of any alleged infringement from time to time.

12.7 UON grants to each Collaborating Organisation a non-exclusive, non-transferable, royalty-free licence to use the Project Intellectual Property:

a. during the Term to the extent necessary to fulfill its obligations under this Agreement in relation to the conduct of the Project and for no other purpose; and

b. for Research Purposes.

12.8 The licence granted in clause 12.7 is conditional upon:

a. the Collaborating Organisation not being in breach of its obligations under this Agreement;

b. Project Intellectual Property being kept confidential by the Collaborating Organisations and otherwise treated as 'Confidential Information' of UON in accordance with clause 10; and

c. the Collaborating Organisations using their best endeavours to protect the Project Intellectual Property including in relation to unauthorised access or use and immediately informing UON of any suspected unauthorised use and taking any action to protect the Project Intellectual Property required by UON.

12.9 For the avoidance of doubt, the licence granted pursuant to clause 12.7 does not confer a right to sub-licence or to Commercialise the Project Intellectual Property.

12.10 UON provides no warranty or assurance as to the suitability of the Project Intellectual Property, Background Intellectual Property or Project results for any purpose and will not be liable for any loss or damage however caused suffered or incurred by a Party and will not, under any circumstances, be liable to any other Party by way of indemnity or by reason of any breach of contract or statutory duty or by reason of tort (including but not limited to negligence) for any loss of profit, loss of use, loss of production, loss of contracts or for any other indirect or consequential damage whatsoever that may be suffered by the other.

13 Moral Rights

13.1 The Parties will respect that UON personnel and students working on the Project may have Moral Rights in the Project Intellectual Property. For the purpose of this clause 13.1, the reasonableness or otherwise of respecting the Moral Rights of the creators of the Project Intellectual Property will be determined by reference to the factors set out at sections 195AR(2) and 195AS(2) of the *Copyright Act 1968* (Cth).

13.2 Each Collaborating Organisation represents and warrants that:

a. the author of any Project Intellectual Property has given, or will give, written consent to the Specified Acts (whether occurring before or after the consent is given) which extends directly or indirectly to the performance of the Specified Acts by UON or any person claiming under or through UON;

b. the author of any Background Intellectual Property has given, or will give, a written consent to the Specified Acts (whether occurring before or after the consent is given) which extends directly and indirectly for UON's benefit in relation to UON's licensed use of such Background Intellectual Property; and

c. it will provide a copy of each such consent to UON upon request.

14 Right to Commercialise

14.1 Subject to clause 14.2:

a. UON as absolute owner of Project Intellectual Property may Commercialise some or all of the Project Intellectual Property or license a Collaborating Organisation or a third party to Commercialise the Project Intellectual Property in its absolute discretion; and

b. each Collaborating Organisation acknowledges and agrees that it has no right, title or interest in any proceeds, profit or similar return and no ownership interest (vested, legal, beneficial or otherwise) if UON elects to Commercialise the Project Intellectual Property.

14.2 To the extent that the Background Intellectual Property of a Collaborating Organisation:

a. forms part of Project Intellectual Property; or

b. is not severable from Project Intellectual Property,

so as to not permit UON to Commercialise the Project Intellectual Property without use of the Background Intellectual Property, then the Collaborating Organisation must negotiate in good faith with UON to agree the terms of a licence of the Background Intellectual Property to allow UON to Commercialise the Project Intellectual Property.

14.3 The terms of the licence referred to in clause 14.2 must be on reasonable terms and conditions and at least on terms and conditions no less favourable than those that the Collaborating Organisation would offer to any third party in an arm's length transaction.

14.4 If negotiations for the terms of the licence referred to in clause 14.2 fail or are not completed within a reasonable time (as determined by UON acting reasonably), UON may refer the matter for determination by an appropriately qualified independent expert chosen by UON (acting reasonably) whose determination will be final and binding unless UON elects that it no longer wishes to acquire the licence of Background Intellectual Property from the Collaborating Organisation.

14.5 Without limitation, the negotiations for the terms of the licence referred to in clause 14.2 must take account of:

a. the respective contributions of UON and the Collaborating Organisation to the development of the Project Intellectual Property and to the Project generally;

b. the degree to which Background Intellectual Property forms part of the Project Intellectual Property; and

c. the inherent market value of the Background Intellectual Property distinct and severable from the Project Intellectual Property.

14.6 If a Collaborating Organisation proposes to Commercialise Project Intellectual Property, it must submit a detailed proposal for that commercialisation to UON for approval (which UON may withhold in its absolute discretion).

14.7 If UON does not approve the proposal from a Collaborating Organisation to Commercialise the Project Intellectual Property, the Collaborating Organisation must not Commercialise the Project Intellectual Property.

14.8 If UON approves the proposal from a Collaborating Organisation to Commercialise the Project Intellectual Property in principal, the Collaborating Organisation must enter into negotiations for a licence permitting the Collaborating Organisation to Commercialise the Project Intellectual Property with:

a. UON regarding the terms and conditions of the use of the Project Intellectual Property and any of its Background Intellectual Property that is necessary in order for the Collaborating Organisation to Commercialise the Project Intellectual Property; and

b. with each other Collaborating Organisation regarding the terms and conditions of the use of any Background Intellectual Property belonging to that Collaborating Organisation that is necessary in order for the Collaborating Organisation to Commercialise the Project Intellectual Property.

14.9 For the avoidance of doubt the fact that a Collaborating Organisation has submitted a proposal to Commercialise Project Intellectual Property as referred to in clause 14.6, or negotiations referred to in clause 14.8 have failed, does not limit or otherwise fetter UON's absolute right to Commercialise the Project Intellectual Property unless and until a duly executed licence agreement between UON and the Collaborating Organisation as referred to in clause 14.8a provides otherwise (if applicable).

15 Infringement

15.1 Each Party will promptly notify the other Parties of:

a. any claim or allegation that the exercise of any rights granted under this Agreement or the use of any Project Intellectual Property or Background Intellectual Property constitutes an infringement of the rights of any third party, and

b. any third party's infringement or threatened infringement of any Project Intellectual Property and Background Intellectual Property of which it becomes aware.

16 Publication

16.1 A Party will not publish any material (papers, articles, presentations or similar disclosures) relating to the Project if that publication will or has the potential to disclose Confidential Information, Background Intellectual Property or Project Intellectual Property without the consent of the other parties (which will not be unreasonably withheld).

16.2 A Party intending to publish will forward a draft of the proposed publication to each other Party not less than 30 days prior to publication.

16.3 A Party may (acting reasonably) oppose publication or require amendments if there are reasonable grounds to believe that publication would adversely affect the protection of Background Intellectual Property, Confidential Information or Project Intellectual Property owned by the Party or a third party.

16.4 If a Party does not advise its opposition to publication within 30 days of its receipt, it will be deemed to have approved the publication unless the Party is aware (or should have been aware) that the publication would compromise the protection of Background Intellectual Property or Project Intellectual Property belonging to UON.

16.5 A Party will not use the name of another Party in any publication, press statement, press conference or other form of publicity without obtaining the prior written consent of the Party provided that such consent will not be unreasonably withheld.

16.6 Any publication must comply with the requirements of the Funding Agreement including the requirement to acknowledge (wherever possible) ARC's contribution and support of the Project.

16.7 The Parties agree that any restriction on the publication of any thesis produced by a student working on the Project will be limited to the minimum restrictions reasonably necessary to protect Confidential Information, Background Intellectual Property and Project Intellectual Property.

17 Project Assets

17.1 Unless otherwise agreed in writing between the Parties, all Project Assets will be the property at all times of and will vest in UON notwithstanding any of those Project Assets may have been purchased with Collaborator Cash Contributions.

17.2 If any of the Project Assets are to be retained by, or returned to, the Collaborating Organisations, UON will be entitled to recover from the Collaborating Organisations, any costs associated with maintaining the Project Assets during the course of the Project.

18 Students, Consultants and Independent Contractors

18.1 Each Party will ensure that any Post Graduate Research Students, independent contractors and/or consultants engaged in, on or in relation in any

way to the Project execute, prior to commencing work, appropriate documentation agreeing to:

a. assign to the Parties, in a manner consistent with the provisions of this Agreement, all Intellectual Property developed by the student, contractor or consultant in relation to the Project (other than copyright in any thesis); and

b. keep any Confidential Information confidential and not to disclose it to any other party and only to use the Confidential Information for the purpose of carrying out work on the Project and for no other purpose.

19 Indemnity

19.1 Each Collaborating Organisation unconditionally and irrevocably releases and indemnifies UON, its officers, employees and agents against any liability, loss, damage, costs and expenses arising from any claim, suit, demand, action or proceeding by any person whether at common law, in equity or pursuant to statute or otherwise, in respect of any loss, death, injury, illness or damage (whether personal or property) arising directly or indirectly:

a. in respect of any infringement (or alleged infringement) of Intellectual Property rights by the Collaborating Organisation, its employees, agents, subcontractors in the course of, or incidental to, performing the Project or the use by UON of any material (including Background Intellectual Property) supplied by the Collaborating Organisation under this Agreement; or

b. where such loss or liability was caused by a wilful, unlawful or negligent act or omission of the Collaborating Organisation, its employees, agents or subcontractors in connection with this Agreement or the Project; or

c. as a result of a breach of this Agreement (including a breach of a warranty provided by a Collaborating Organisation) or the Funding Agreement.

20 Insurance

20.1 Each Collaborating Organisation must effect and maintain adequate insurance (including public liability and professional indemnity insurance) to cover any liability arising as a result of its participation in the Project and, if requested, provide UON with a copy of the relevant policies or such other evidence satisfactory to UON. Each Collaborating Organisation will be also responsible for effecting all insurances required under workers' compensation legislation and for taking all action required as an employer.

21 Dispute Resolution

21.1 A Party to this Agreement will not start arbitration or court proceedings (except proceedings seeking interlocutory relief) in respect of a dispute arising out of this Agreement ("dispute") unless that Party has complied with this clause 21.

21.2 A Party claiming that a dispute has arisen must notify the other Parties of the dispute and give details of the dispute.

21.3 During the twenty one (21) days after the notice of dispute is served (or such longer period as may be agreed in writing by the Parties to the dispute) each Party to the dispute will use its best endeavours to resolve the dispute.

21.4 If the Parties are unable to resolve the dispute within the twenty one (21) days after the notice is served, the Parties agree that any Party may refer the dispute for mediation in accordance with the mediation rules of the Law Society of New South Wales, to:

a. a mediator agreed by the Parties; or

b. if the Parties are unable to agree on a mediator within seven (7) days after the end of the period agreed to under sub-clause 21.4, a mediator nominated by the President for the time being of the Law Society of New South Wales.

21.5 The Parties will request the mediator to assist in negotiating a resolution of the dispute.

21.6 A Party may agree in writing to a mediator making a decision that is binding on that Party.

21.7 The Parties will keep confidential, and will not use, any information or documents disclosed by a Party under this clause 21 except to attempt to resolve the dispute.

21.8 Each party will bear its own costs of complying with this clause 21 and the Parties will bear equally the costs of any mediator engaged.

22 Termination

22.1 This Agreement will commence on Commencement Date and will continue in force until the expiry of the Term unless UON earlier terminates this Agreement by giving the Collaborating Organisations 30 days' prior written notice (or such lesser period provided elsewhere in this Agreement).

22.2 Subject to the dispute resolution procedures set out in clause 21, a Party may terminate this Agreement by written notice to the other Parties, if a Party is in breach of its obligations under this Agreement and it fails to remedy such breach, or take effective action to do so, within 21 days of a written request or if in the reasonable opinion of the Party the breach is not capable of remedy.

22.3 UON may, without penalty, immediately terminate this Agreement if:

a. an Insolvency Event occurs in respect of a Collaborating Organisation;

b. the ARC Funds are withdrawn, withheld or otherwise varied;

c. the Funding Agreement is terminated;

d. a Collaborating Organisation notifies UON that it is unable or unwilling to proceed or continue with the Project including the cessation of a Chief Investigator or Partner Investigator (as the case may be);

e. UON is unable or unwilling to proceed or continue with the Project; and

f. UON is otherwise required to terminate the Project.

22.4 Termination of this Agreement will be without prejudice to any obligation by any Party to any other Party which has already accrued.

23 Notices

23.1 The addresses for notices are set out in Item 9 of Schedule 1.

24 Waiver

24.1 A waiver by a Party in respect of any particular breach of a condition or provision of this Agreement will not be deemed to be a waiver in respect of any other or subsequent breach.

25 GST

25.1 Notwithstanding any other provision of this Agreement if GST applies to any supply made by a Party in connection with this Agreement, the consideration provided or to be provided for that supply will be increased by an amount equal to the GST liability properly incurred by the Party making the supply.

25.2 Each Party warrants that at the time any supply is made under this Agreement on which GST is imposed, the Party is or will be registered under GST Law. If a Party requests written evidence of the registration of another Party, that other Party will promptly produce evidence satisfactory to the Party seeking the evidence.

25.3 Any invoice rendered by a Party in connection with a supply under this Agreement which seeks to recover an amount of GST payable by that Party must conform to the requirements for a tax invoice as set out in the GST Law. If requested to do so by the recipient of the supply, the supplier must provide a tax invoice within 14 days.

25.4 For the purposes of this clause, "GST", "Tax Invoice" and "Supply" have the meanings attributed to those terms in the GST Law.

26 Entire Agreement and Variation

26.1 This Agreement (and to the extent content is incorporated or derived from it, the Funding Agreement) constitutes the entire agreement between the Parties and supersedes all communications, negotiations, arrangements and agreements, either oral or written, between the Parties with respect to the subject matter of this Agreement.

26.2 No agreement or understanding varying or extending this Agreement, including in particular the scope of the Project will be legally binding upon the Parties unless in writing and signed by all Parties.

27 General

27.1 Any provisions of this Agreement which are held to be illegal or otherwise in conflict with any laws, statutes or regulations will be deemed to be severed from the remainder of the Agreement and the validity of the remaining provisions will not be affected.

27.2 UON may sub-contract or assign its rights under this Agreement or the performance of any part of the Project provided this is not in breach of the Funding Agreement. A Collaborating Organisation must not sub-contract or assign its rights under this Agreement or the performance of any part of the Project without UON's prior written consent (which it may withhold in its absolute discretion).

27.3 This Agreement does not establish a partnership between the Parties and no Party may act on behalf of another Party or bind another Party to any legal obligation.

27.4 The rights and obligations contained in clauses 2, 3, 5, 6, 7, 10, 11, 12, 13, 14, 16, 17, 19, 20 and this clause 27 survive termination of this Agreement.

27.5 This Agreement is governed by the laws of New South Wales and each party submits to the jurisdiction of the courts of that place and its courts of appeal.

27.6 A Party will not be liable for any failure to carry out its obligations under this Agreement where such failure is due to an event of Force Majeure provided that the Party acts in good faith and uses its best endeavours to comply with its obligations under this Agreement.

27.7 This Agreement may be executed in any number of counterparts.

27.8 The Parties will bear their own costs and expenses in relation to the negotiation, preparation, execution, delivery and completion of this Agreement and any other related documentation.

EXECUTED BY THE PARTIES

EXECUTED for and on behalf of **THE UNIVERSITY OF NEWCASTLE** ABN 15 736 576 735 by its authorised officer:

Signature

Witness

MISS JODIE BROWN
Personal Assistant to the Deputy Vice-Chancellor (Research)

Print Name

PROFESSOR NICHOLAS TALLEY
~~ACTING DEPUTY VICE-CHANCELLOR~~ (RESEARCH)

Print Name

Office Held

EXECUTED for and on behalf of **HUNTER NEW ENGLAND LOCAL HEALTH DISTRICT** ABN 63 598 010 203 by its authorised officer:

K. P. Kelly

Signature

K.E. Burns

Witness

K. P. KELLY

Print Name

Kay BURNS

Print Name

EA, Director N+M Services.

DIRECTOR CLINICAL SERVICES,

Office Held

NURSING & MIDWIFERY
HNE LHD. 6/02/2014.

Schedule 1

Item 1: **Project**

Moving from policy to practice: A randomised trial of an implementation intervention to facilitate the adoption of a statewide healthy canteen policy

Item 2: **Eligible Organisations**

Not Applicable

Item 3: **Partner Organisations**

Hunter New England Local Health District ABN 63 598 010 203 of Lookout Road, Lambton, NSW 2305

Item 4: **Chief Investigators**

UON: Dr Luke Wolfenden
Professor John Wiggers
Associate Professor Andrew Bell
Dr Megan Freund

Item 5: **Partner Investigators**

HNELHD: Ms Karen Gillham
Dr Elizabeth Campbell
Ms Rachel Sutherland
Ms Nicole Nathan

Item 6: **Term**

2 years

Item 7: **Project Sunset Date**

30 June 2014

Item 8: **Background Intellectual Property**

Pre-existing resources and intellectual property developed as part of the Hunter New England Good for Kids, Good for Life initiative.

Item 9: **Address for Notices**

UON:

Attn: Lyn McBriarty, Director, Research Services
University of Newcastle
University Drive, Callaghan NSW 2308
Telephone: (02) 4921 5300
Fax: (02) 4921 7164
Lyn.McBriarty@newcastle.edu.au

HNELHD:

Attn. Ms Karen Kelly
Director Clinical Services, Nursing and Midwifery / HSFAC
Hunter New England Local Health District
Locked Bag 1
New Lambton NSW 2305
Telephone: (02) 4921 4915
Fax: (02) 4921 4939
Karen. Kelly@hnehealth.nsw.gov.au

Schedule 2
Project Contributions

ARC Support (excluding GST):

	2013	2014	2015
Cash	$95,763.50	$19,5057.00	$99,293.50
In-kind	$0.00	$0.00	$0.00
TOTAL	$95,763.50	$19,5057.00	$99,293.50

UoN Support (excluding GST) (in addition to ARC Support)

	2013	2014	2015
Cash	$0.00	$0.00	$0.00
In-kind	$6,311	$10,936.50	$4,625.50
TOTAL	$6,311	$10,936.50	$4,625.50

HNELHD Support (excluding GST):

	2013	2014	2015
Cash	$34,216.50	$63,999.00	$29,782.50
In-kind	$118,534.00	$240,032.00	$121,498.00
TOTAL	$152,750.50	$304,031.00	$151,280.50

Annexure A

2013 ARC Linkage Projects Scheme – Proposal

Annexure B

2013 ARC Linkage Projects Scheme – Funding Agreement
