# Supplementary material for: A randomised controlled trial of an intervention to increase the implementation of a healthy canteen policy in Australian primary schools: study protocol
Source: Implement Sci. 2014 Oct 11;9:147. doi: 10.1186/s13012-014-0147-3 (PMC4197283; doi:10.1186/s13012-014-0147-3)
Supplement: Additional file 2: — Ethics approval from Hunter New England Human Research Ethics Committee. [file 13012_2014_147_MOESM2_ESM.pdf]

NSW GOVERNMENT | Health
Hunter New England
Local Health District

15 July 2013

Dr L Wolfenden
Director
Population Health
Wallsend Campus

Dear Dr Wolfenden

Re: HNE Kids Healthy Eating and Physical Activity Program (06/07/26/4.04)

Thank you for submitting a request for an amendment to the above project. This amendment was reviewed by the Hunter New England Human Research Ethics Committee. This Human Research Ethics Committee is constituted and operates in accordance with the National Health and Medical Research Council's *National Statement on Ethical Conduct in Human Research (2007)* (National Statement) and the *CPMP/ICH Note for Guidance on Good Clinical Practice*. Further, this Committee has been accredited by the NSW Department of Health as a lead HREC under the model for single ethical and scientific review.

I am pleased to advise that the Hunter New England Human Research Ethics Committee has granted ethical approval for the following amendment requests:

- For the addition of Dr Megan Freund as co-investigator;
- To link an Australian Research council (ARC) funded grant to the Good for Kids Program; and
- To register the title of the Australian Research Council (ARC) funded grant *"Moving from policy to practice: A randomised trial of an implementation intervention to facilitate the adoption of a state-wide health canteen policy"* to the Good for Kids Program

For the protocol**: HNE Kids Healthy Eating and Physical Activity Program**

Approval has been granted for this study to take place at the following site:

- **Hunter New England Local Health District**

Approval from the Hunter New England Human Research Ethics Committee for the above protocol is given for a maximum of **5** years from the date of the approval letter of your initial application after which a renewal application will be required if the protocol has not been completed. The above protocol is approved until **November 2016.**

Hunter New England Human Research Ethics Committee
(Locked Bag No 1)
(New Lambton NSW 2305)
Telephone (02) 49214 950 Facsimile (02) 49214 818
Email:hnehrec@hnehealth.nsw.gov.au
http://www.hnehealth.nsw.gov.au/research_ethics_and_governance_unit

The *National Statement on Ethical Conduct in Human Research (2007)* which the Committee is obliged to adhere to, include the requirement that the committee monitors the research protocols it has approved. In order for the Committee to fulfil this function, it requires:

- A report of the progress of the above protocol to be submitted at 12 monthly intervals. Your review date is **November 2013.** A proforma for the annual report will be sent two weeks prior to the due date.

- A final report must be submitted at the completion of the above protocol, that is, after data analysis has been completed and a final report compiled. A proforma for the final report will be sent two weeks prior to the due date.

- All variations or amendments to this protocol, including amendments to the Information Sheet and Consent Form, must be forwarded to and approved by the Hunter New England Human Research Ethics Committee prior to their implementation.

- The Principal Investigator will immediately report anything which might warrant review of ethical approval of the project in the specified format, including:

    - any serious or unexpected adverse events

        - Adverse events, however minor, must be recorded as observed by the Investigator or as volunteered by a participant in this protocol. Full details will be documented, whether or not the Investigator or his deputies considers the event to be related to the trial substance or procedure.

        - Serious adverse events that occur during the study or within six months of completion of the trial at your site should be reported to the Professional Officer of the Hunter New England Human Research Ethics Committee as soon as possible and at the latest within 72 hours.

        - Copies of serious adverse event reports from other sites should be sent to the Hunter New England Human Research Ethics Committee for review as soon as possible after being received.

        - Serious adverse events are defined as:

            - Causing death, life threatening or serious disability.
            - Cause or prolong hospitalisation.
            - Overdoses, cancers, congenital abnormalities whether judged to be caused by the investigational agent or new procedure or not.

    - Unforeseen events that might affect continued ethical acceptability of the project.

- If for some reason the above protocol does not commence (for example it does not receive funding); is suspended or discontinued, please inform Dr Nicole Gerrand, the Manager, Research Ethics and Governance Unit as soon as possible.

The Hunter New England Human Research Ethics Committee also has delegated authority to approve the commencement of this research on behalf of the Hunter New England Local Health District. This research may therefore commence.

Should you have any queries about your project please contact Dr Nicole Gerrand as per the contact details at the bottom of the page. The Hunter New England Human Research Ethics Committee Terms of Reference, Standard Operating Procedures, membership and standard forms are available from the Hunter New England Local Health District website:
Internet address: http://www.hnehealth.nsw.gov.au/research_ethics_and_governance_unit

Please quote **06/07/26/4.04** in all correspondence.

The Hunter New England Human Research Ethics Committee wishes you every success in your research.

Yours faithfully

For: Professor M Parsons
Chair
Hunter New England Human Research Ethics Committee

Hunter New England Human Research Ethics Committee
(Locked Bag No 1)
(New Lambton NSW 2305)
Telephone (02) 49214 950 Facsimile (02) 49214 818
Email:hnehrec@hnehealth.nsw.gov.au
http://www.hnehealth.nsw.gov.au/research_ethics_and_governance_unit
